# Dissecting Shared Genetic Architecture of Thoracic Aortic Aneurysm and Aortic Related Traits and Identifying SplA/Ryanodine Receptor Domain and SOCS Box Containing 1 Involved in Smooth Muscle Phenotype Switching and Cell Senescence Through Alternative Splicing

**DOI:** 10.1096/fj.202502457R

**Published:** 2025-11-18

**Authors:** Qingyang Song, Tongxin Chu, Quan Liu, Rennan Weng, Huayang Li, Mengya Liang, Zhongkai Wu

**Affiliations:** ^1^ Department of Cardiac Surgery First Affiliated Hospital of Sun Yat‐Sen University Guangzhou China; ^2^ Department of Cardiology Fuwai Hospital Chinese Academy of Medical Sciences Shenzhen China

**Keywords:** alternative splicing, cell senescence, genome‐wide association study (GWAS), phenotype switching, smooth muscle cell, SplA/ryanodine receptor domain and SOCS box containing 1 (SPSB1), thoracic aortic aneurysm (TAA)

## Abstract

Machine learning was employed to annotate images of the thoracic aorta, resulting in the identification of additional thoracic‐related targets. However, genetic evaluation of the associations among thoracic aortic traits and their shared genes is lacking. We integrated thoracic aortic aneurysm (TAA) genome‐wide association study (GWAS) data and genetically related traits' GWAS to identify the shared genetic variants with multi‐trait analysis of GWAS (MTAG) and N‐weighted GWAMA (N‐GWAMA). The robust shared gene, SplA/Ryanodine Receptor Domain and SOCS Box Containing 1 (SPSB1), was validated in TAA mouse models and TAA patients. Single‐cell RNA sequencing was used to identify the specific cell niches. Additionally, functional experiments were implemented to examine the function of SPSB1 in smooth muscle cell (SMC) phenotypic transformation. Mass spectrometry and RNA sequencing were used to identify the potential function of SPSB1. We revealed a strong relationship between TAA, thoracic aortic diameter, and thoracic aortic area. 3091 shared variants in 50 loci reached significance (*p* < 5 × 10^−8^) in MTAG and N‐GWAMA analysis. By integrating transcriptomics data and in vivo experiments, only SPSB1 was verified as a robust target. Single‐cell RNA sequencing identified SPSB1 as a major regulator of SMC phenotype switching in TAA. In vitro, SPSB1 silencing inhibited SMCs toward a synthetic phenotype and cell senescence. Mass spectrometry and RNA sequencing indicated SPSB1 is involved in alternative splicing within SMC. We dissected the shared genetic architecture among TAA, thoracic aortic diameter, and thoracic aortic area. SPSB1 was the most robust shared target. Functional analyses showed SPSB1 may play a role in SMC phenotype switching and cell senescence through alternative splicing. These discoveries will offer novel insights into the pathogenesis of TAA.

## Graphical abstract

The flowchart of the study. BAPN, β‐aminopropionitrile; FUMA, Functional Mapping and Annotation; GO, Gene Ontology; GWAS, genome‐wide association study; KEGG, Kyoto encyclopedia of genes and genomes; LDSC, LD score regression; MR‐JTI, mendelian randomization joint‐tissue imputation; MTAG, multi‐trait analysis of GWAS; siRNA, small interfering RNA; SPSB1, SPRY Domain‐Containing SOCS Box Protein 1; TAA, thoracic aortic aneurysm.

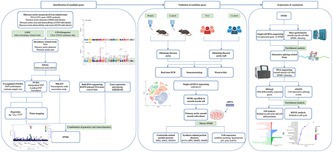

**Abbreviations**
A3SS: alternative 3′ splice site
A5SS: alternative 5′ splice site
AAdiameter: ascending thoracic aortic diameter
AAdis: ascending thoracic aortic distensibility
AAmax: ascending thoracic aortic max area
AAmin: ascending thoracic aortic minimum area
AAstrian: ascending thoracic strain
AS: alternative splicing
ASAP: Advanced Study of Aortic Pathology study
ASE: alternative splicing events
BAPN: β‐aminopropionitrile
CTI: cross‐trait LD‐score intercepts
CXCL: chemokine (C‐X‐C motif) ligand
DAdiameter: descending thoracic aortic diameter
DAdis: descending thoracic aortic distensibility
DAmax: descending thoracic aortic max area
DAmin: descending thoracic aortic minimum area
DAS: differentially alternative splicing events
DAstrain: descending thoracic aortic strain
DEG: differentially expressed gene
DIA: data‐independent acquisition
ELISA: enzyme‐linked immunosorbent assay
FUMA: Functional Mapping and Annotation
GEM: gene expression microarray
GEO: Gene Expression Omnibus
GO: Gene Ontology
GWAS: genome‐wide association study
IL: interleukin
KEGG: Kyoto encyclopedia of genes and genomes
LDSC: linkage disequilibrium score regression
MAF: minor allele frequency
maxFDR: maximum false discovery rate
MCP: monocyte chemoattractant protein
MMP: matrix metalloproteinase
MR‐JTI: Mendelian randomization framework for causal inference
MTAG: Multi‐trait analysis
MXE: mutually exclusive exon
N‐GWAMA: N‐weighted GWAMA
*P*_A_
: variant specific alignment prior
PP: posterior probability
*P*_R_
: variant specific regional prior
PSI: percent‐spliced‐in
qPCR: real‐time quantitative polymerase chain reaction
*r*_g_
: genetic correlation
RI: retained intron
SA‐β‐gal: Senescence‐associated β‐galactosidase
scRNA‐seq: single‐cell RNA sequencing
SE: skipped exon
SMC: smooth muscle cell
SNP: single nucleotide polymorphism
SPSB1: SplA/Ryanodine Receptor Domain and SOCS Box Containing 1
TAA: Thoracic aortic aneurysm
TAC: transverse aortic constriction
UKBB: United Kingdom Biobank
WB: Western Blot

## 1 Introduction

A thoracic aortic aneurysm (TAA) represents a localized dilation that involves the aortic root, ascending aorta, aortic arch, or descending aorta. TAA is frequently asymptomatic in its normal course and is usually discovered by chance when imaging is done for consequences like rupture or dissection [1]. Currently, treatment options are largely limited to surgical intervention, as no effective pharmacological therapies exist to prevent aneurysm progression or rupture—particularly in non‐Marfan syndrome TAA patients [2]. In addition, the lack of reliable methods for identifying high‐risk TAA cases makes it a challenge to reduce mortality rates through early surgical intervention [3]. Approximately 20% of TAAs are attributable to specific genetic variants, offering an opportunity to identify druggable targets and biomarkers via genetic risk‐based screening [4]. Several genes, including *ACTA2*, *LOX*, *PI15*, *ELN*, *FLNB*, *ULK4*, *FBN1*, *PRDM6*, *THSD4*, and *MASP1*, have been associated with increased TAA risk [5]. However, genetic research has traditionally focused on familial cases [5]. Moreover, the sensitivity of traditional case–control genome‐wide association studies (GWAS) to fully capture the genetic architecture of TAA is low [6]. Furthermore, the diameter threshold of TAA remains controversial, which makes it hard to reveal regulatory mechanisms driving TAA [3].

With the machine learning to annotate images of thoracic aorta, more thoracic traits can be measured and used as phenotypes in GWAS. The use of novel analytical approaches enhances the statistical power to identify thoracic aortic‐related genetic variants [6]. Additionally, multi‐trait joint analysis leverages correlation information from genetically related traits to increase discovery efficiency [7, 8]. Integrating multiple thoracic aortic traits using robust statistical tools further improves the sensitivity and power of GWAS targeting individual thoracic traits. The focus of the study turned out to discover new targets for therapy to TAA through cross‐trait meta‐analysis of genetically correlated thoracic aortic traits. By integrating genomic and transcriptomic data, we prioritized candidate genes. In vivo, SplA/Ryanodine Receptor Domain and SOCS Box Containing 1 (SPSB1) expression was significantly elevated in TAA mouse models and patients. In vitro, SPSB1 was found to regulate smooth muscle cell phenotype switching and senescence via alternative splicing, suggesting a potential role in TAA pathogenesis.

## 2 Materials and Methods

### 2.1 Study Design

Graphical abstract displays the study's flowchart. First, we integrated genome‐wide association study (GWAS) data on genetically correlated traits (genetic correlation, *r*_g_) of thoracic aortic aneurysm (TAA) from the UK Biobank (UKBB) (Table S1). We then incorporated transcriptomic data and found potential genes that are associated with TAA. These genes were subsequently validated in two distinct murine TAA models and in human TAA patients. Using single‐cell RNA sequencing (scRNA‐seq) to analyze human thoracic aorta tissue, we identified the cellular niches associated with the gene and silenced it in primary cells to investigate its function. Finally, we employed quantitative proteomics and RNA sequencing (RNA‐seq) in gene‐knockdown primary cells to elucidate the gene's potential mechanism.

### 2.2 GWAS Summary Statistics Quality Evaluation

GWAS summary data limited exclusively to individuals of European ancestry to comply with the quality control procedures previously described (Table S1) [9, 10, 11, 12]. A filter was applied to each dataset to exclude SNPs with a minor allele frequency (MAF) of under 0.01 and SNPs alongside absent or duplicated rsID. Allele harmonization was performed using GWASinspector, with the 1000 Genomes Project Phase 3 (European ancestry) GRCh37 (hg19) reference panel [13, 14].

### 2.3 Genetic Correlation and Heritability

The heritability and genetic correlation of complex variables were estimated using linkage disequilibrium score regression (LDSC) [15, 16]. For a calculation of the variance in TAA and aortic traits explained by SNPs in individuals of European ancestry, we initially conducted single‐trait LDSC. LDSC assumes that the cumulative effect for all SNPs in linkage disequilibrium (LD) linked to a SNP is captured by its effect. The effect sizes of SNPs with higher LD scores are higher because they are more likely to capture causal variants than those that identify a limited number of markers. Heritability estimates were derived by regressing effect sizes against LD scores. The liability scale was used to convert SNP heritability estimates for TAA, as determined by the observed sample and population prevalence (0.16%) [17].

In order to estimate genetic correlations (*r*_g_) between TAA and other aortic characteristics (e.g., thoracic aortic diameter, area, distensibility, and strain), we conducted pairwise LDSC using pre‐computed LD scores from the 1000 Genomes Project Phase 3 (European ancestry) dataset [18]. In order to alleviate the effects of poor imputation quality, we limited the analysis to HapMap3 SNPs that were well imputed. The significance threshold was established at *p* < 9.09 × 10^−4^ (0.05/55) using the Bonferroni correction. Only aortic traits with strong correlations (*p* < 1.82 × 10^−4^) to TAA were considered for further analysis.

### 2.4 Cross‐Trait Meta‐Analysis With MTAG and N‐GWAMA

A technique known as the multi‐trait analysis of GWAS (MTAG) employs generalized inverse‐variance‐weighted meta‐analysis to investigate numerous correlated characteristics. It is designed to enhance the statistical power of each trait in utilizing the connections among correlated traits to identify new genetic associations [7]. This approach employs summary statistics coming from single‐trait GWAS to generate trait‐specific association statistics. The input summary statistics do not require independent discovery samples, as MTAG employs LDSC to compensate for the (potentially unknown) sample overlap that exists among GWAS results in specific features. The main assumption of MTAG is that there is a homogeneous variance–covariance matrix of effect sizes for all SNPs across the variables. The estimator of MTAG can, however, maintain its consistency even if the previous assumption falls short when specific SNPs affect just a subset of the characteristics. We limited our primary analysis to TAA, thoracic aortic diameter, and thoracic aortic area due to the increased number of shared risk loci and stronger genetic correlations between TAA and numerous aortic characteristics. We employed the summary statistics of TAA and the aortic characteristics that were genetically related to it as input, along with the total statistics about TAA coming from MTAG analysis for MTAG‐TAA. The codes to perform these analyses were available from GitHub (https://github.com/JonJala/mtag).

N‐weighted GWAMA (N‐GWAMA) quantifies dependencies between summary statistics using pairwise cross‐trait LD‐score intercepts (CTI) [8]. The CTI is similar to the covariance between the test statistics of the traits between two univariate GWASs. This method is robust to overlapping sample datasets and estimates effect sample sizes based on CTI. N‐GWAMA analysis was performed to verify MTAG's significant genetic associations and to improve power for discovering additional variants. SNPs with both *P*_*MTAG*_ and *P*_*N‐GWAMA*_ less than 5 × 10^−8^ had been determined to require additional verification. The effective sample size could be estimated from the R script *N_weighted_GWAMA.function.R* is available from GitHub (https://github.com/baselmans/multivariate_GWAMA).

### 2.5 SNP Annotation Using FUMA

The genome‐wide significant SNPs of TAA GWAS and MTAG‐TAA were annotated using Functional Mapping and Annotation (FUMA) v1.5.2 [19], an online platform available at https://fuma.ctglab.nl/. We employed the 1000 Genomes Project Phase 3 of European ancestry as a reference panel and conducted FUMA annotation with default parameters. Independent significant SNPs were defined as SNPs with *p* < 5 × 10^−8^ and independent from each other at *r* ^2^ < 0.6 within 1 Mb. Lead SNPs, a subset of the independent significant SNPs, were defined as those that are independent from each other at *r* ^2^ < 0.1. Independent significant SNPs in LD blocks that are located less than 250 kb apart were integrated to pinpoint genomic risk loci. In addition, FUMA annotated genome‐wide significant SNPs from GWAS summary statistics of TAA for comparison.

### 2.6 Multi‐Trait Colocalization and Bayesian Fine‐Mapping Analysis

The genetic etiology of shared attributes is assessed through the use of statistical colocalization. In order to efficiently assess colocalization across multiple characteristics employing summary statistics, we implemented HyPrColoc [20], a Bayesian algorithm. In order to further colocalize causal variants across TAA, TAA diameter, and TAA area, multi‐trait colocalization analysis took place for every SNP associated with each locus identified through FUMA. The default variant‐specific regional and alignment priors (*P*_R_ = *P*_A_ = 0.5) were used to determine the evidence for colocalization. Colocalization was identified when PR × PA ≥ 0.25 [19]. The LocusZoom plots were used to display the verified locus [20]. Additionally, Bayesian fine‐mapping analysis has been used to produce SNP credible sets on every locus that was examined in the HyPrColoc analyses.

### 2.7 Gene Microarray

The source of the gene expression microarray was the Advanced Study about Aortic Pathology (ASAP) study [21]. Gene Expression Omnibus (GEO) provided the microarray dataset under accession number GSE26155 to validate the expression of candidate causal TAA genes which were discovered in SNP‐based or gene‐based analysis. The samples with an aortic diameter > 4.5 cm were defined as the TAA group and aortic diameter < 4 cm defined as the normal group [3]. Only the samples obtained from the aorta intima‐media of patients who have a tricuspid aortic valve were incorporated into this research. Finally, 22 TAA samples and 31 normal samples were obtained for further analysis. Two‐tailed *t*‐tests were implemented, and Bonferroni corrections were applied.

### 2.8 Single Cell RNA‐Seq Analysis of Human Thoracic Aorta

The scRNA‐seq was obtained from Yanming et al. and consisted of 48 082 cells along with 11 cell types about 8 TAA patients and 3 healthy samples [22]. The R package Seurat (Version 4.0.5) was used for finding pertinent cell types in the individual putative causal TAA risk genes in the dataset, which was accessible from GEO under the accession number GSE155468. The FindAllMarkers function was employed to detect genes that establish clusters. Dimensionality reduction with t‐SNE was performed, and the annotation of each cell cluster corresponded to the original clusters from Yanming et al.

### 2.9 Enrollment of Study Participants and Collection of Thoracic Aortic Tissues

The Ethics Committee of the First Affiliated Hospital of Sun Yat‐Sen University approved the protocol for the collection of human aortic tissue samples ([2018]118). The committee‐approved guidelines were strictly adhered to in all experiments that involved human aortic tissue samples. Families of all participants were granted informed assent. Seven heart transplant recipients provided human aortic samples for control. Human TAA samples have been collected from about 7 individuals whose work was diagnosed with TAA and who received surgical replacement of the ascending aorta. None of the included patients had a heritable form of aortopathy or connective tissue disorders (e.g., Marfan syndrome, Loeys‐Dietz syndrome, bicuspid aortic valve disease), nor was their aortic disease related to infection, aortitis, or trauma. The 2014 ESC Guidelines on the diagnosis and management of aortic diseases were used to diagnose the TAA patients [23]. Samples were obtained from the ascending aorta between the aortic sinotubular junction and the innominate artery of all participants. Table S2 summarizes the clinical characteristics of all participants are available.

### 2.10 Disease Models

The ethics committee of the China Sun Yat‐sen University (SYSU‐IACUC‐2024‐001388) endorsed the guidelines for the care and use of laboratory animals, and they were adhered to in all animal experiments. The Guangdong Gempharmatech Co Ltd provided the C57BL/6J male rodents. The animals were confined in a specific pathogen‐free facility and were subjected to a controlled temperature cycle that alternated between 12 h of light and 12 h of darkness. They were supplied with water and a standard rodent diet. The ethics committee of Sun Yat‐Sen University endorsed the guidelines for the care and use of laboratory animals, which were adhered to in all animal experiments.

For induction of a pharmacological model for thoracic aortic pathology [24], 50 three‐week‐old mice were randomly divided into 2 groups and were fed with 0.25% β‐aminopropionitrile (BAPN) (Cat number A0408, TCI, China) dissolved in water (*n* = 35) or distilled water (*n* = 15) for 4 weeks. For experiments with the BAPN‐treated group, 17 (48.6%) mice ultimately survived. The rodents were sacrificed after being examined with echocardiography. For Western blot and histological analysis, the blood vessels extending from the ascending aorta to the descending branch of the thoracic aorta were collected. Twelve‐week‐old mice were used to establish the transverse aortic constriction (TAC) model for pressure‐overload‐induced ascending aortic aneurysm as reported [25]. Briefly, mice were anesthetized with isoflurane delivered using a vaporizer at 1.5%–2.5% during the surgery. To implement TAC, a 27‐gauge needle was positioned in the arch at the intersection of the brachiocephalic trunk and the left common carotid artery, and a 7–0 silk suture was wrapped around it. Subsequently, the muscles and epidermis were meticulously sutured in a specific order, and the rodents were permitted to awaken. 16 TAC mice and 15 sham‐operated mice were used for follow‐up experiments. Echocardiography was performed on the rodents fourteen days following surgery, and they were sacrificed before the maladaptive cardiac failure that is associated with TAC occurred. For histological and Western blot analyses, the ascending aortas were collected.

### 2.11 Ultrasound Imaging

Ultrasound imaging was conducted with the Vevo 2100 high‐resolution imaging system (FUJIFILM VisualSonics, Toronto, Canada) and an 18‐ to 38‐MHz (MS400, mouse cardiovascular) scan head. In order to reduce heat loss, mice were positioned in a supine position on a homeothermic plate. The investigation was conducted with continuous recording of respiratory rate, pulse rate, and rectal temperature. Mice were positioned in an anesthesia induction chamber, and isoflurane was administered at a concentration of 1.5%–2.5% using a vaporizer for anesthesia. Preheated ultrasound gel was administered to the animals after they were depilated. Whenever feasible, the pulse rates were preserved at 450–500 beats per minute (bpm). The diameter of the ascending aorta in the vicinity of the brachiocephalic artery has been determined at the conclusion of diastole. From each animal, an average of three measurements was taken. All recordings were made by a single expert echocardiographer, who was blinded to treatment.

### 2.12 RNA Extraction, cDNA Synthesis, and Real‐Time PCR

The aorta tissue from mice and humans was subjected to RNA isolation using the Trizol Reagent (15596026, Invitrogen Life Technologies, USA). Total RNA of cells was extracted using the EZ‐press RNA Purification Kit (Cat number B0004D, EZBioscience, USA). The concentration, quality, and integrity were determined using a NanoDrop spectrophotometer (ND‐ONE‐W, Thermo Scientific, USA). The RNA was reverse‐transcribed into cDNA using the First Strand cDNA Synthesis Kit (Cat number EZB‐RT2GQ, EZBioscience, China), according to the manufacturer's instructions. Real‐time quantitative polymerase chain reaction (qPCR) analysis was conducted applying the SYBR Green qPCR Master Mix (ROX1 Plus) (Cat number A0012‐R1, EZBioscience, China) on a LightCycler 480 Instrument II Real‐Time PCR System (Roche, Kaiseraugst, Switzerland). All samples were amplified using 3–8 biological replicates per sample. The comparative CT method was employed to analyze the results. In order to account for variations in cDNA deposition, the samples were normalized to GAPDH. To summarize the primer sequences for PCR, refer to Table S3.

### 2.13 Enrichment Analysis of *SPSB1* Co‐Expressed Genes

In SMC1 of scRNA‐seq, we calculated the Pearson correlation between *SPSB1* and other genes, and *SPSB1* co‐expressed genes were defined by threshold correlation *R* > 0.3 and significance *p* < 0.05. Then we performed Gene Ontology (GO) enrichment analysis for *SPSB1* co‐expressed genes by clusterProfiler R package (Version 4.10.1). Bonferroni test was used as the adjustment method of *p* value.

### 2.14 Western Blot Analysis

The aortas from mice and humans were lysed with lysis buffer and ground with a tissue grinder (LUKYM, China). Aortic SMCs were cultured in 6‐well plates and scraped with 1 × radioimmunoprecipitation assay (RIPA) buffer (Cat number P0013B, Beyotime, China) supplemented with 1% proteinase inhibitor. This was followed by centrifugation of the samples at a speed of 12 000 rpm for 15 min while kept at 4°C. After boiling at 100°C for 10 min, the supernatant was combined with loading buffer and subsequently subjected to SDS‐PAGE. PVDF membranes were utilized to transmit the proteins. Following a 2‐h blocking period at room temperature in TBST buffer containing 5% BSA, the membranes were incubated with a primary antibody (dissolved in primary antibody dilution buffer according to the manufacturer's instructions) at 4°C with moderate agitation overnight. The antibodies used include: anti‐SPSB1 (Cat number A13886, ABclonal, China), anti‐α‐SMA (Cat number 19245s, CST, USA), anti‐MYH11, anti‐SM22, anti‐MMP9 (Cat number GB12132, servicebio, China), anti‐KLF4 (Cat number WL02532, Wanlelbio, China), anti‐OPN (Cat number WL02378, Wanlelbio, China), anti‐β‐tubulin (Cat number AF7011, Affinity, China). Following this, the membranes were washed three times with TBST for 10 min each and incubated with a goat anti‐rabbit antibody (Cat number HS101‐01, TransGen Biotech, China) and a goat anti‐mouse antibody (Cat number HS201‐01, TransGen Biotech, China). Chemiluminescence detection was performed using an HRP substrate (Cat number WBKLS0050, Millipore, China) in accordance with the manufacturer's instructions. Additionally, the secondary antibodies were diluted in 5% BSA in TBST prior to their use. The intensities of the bands in the Western blot were quantified using ImageJ software through densitometry.

### 2.15 Histology and Immunohistochemistry

The aortic tissues of humans and mice were surgically removed, preserved in 4% paraformaldehyde for one night, embedded in paraffin, and cut into 3 μm thick sections. Cross‐sections of mouse arteries were stained with hematoxylin and eosin (H&E). Additionally, Sirius Red staining was employed to assess collagen content within the arterial wall.

For immunostaining, the sections were deparaffinized, retrieved for antigens, and incubated with primary antibodies at 4°C overnight. Hematoxylin was used to counterstain the sections, and secondary antibodies were applied for 60 min at room temperature. The slides were imaged with fluorescence or confocal microscopy. The primary antibodies used included anti‐SPSB1 (orb514966, Biorbyt, UK).

### 2.16 SA‐β‐Gal Staining

Senescence‐associated β‐galactosidase (SA‐β‐gal) activity has been monitored via the SA‐β‐gal Staining Kit (Cat number BL133A, Biosharp, China). Staining solution was incubated at 37°C overnight after aortic SMCs were mounted in 6‐well plates, fixated in a fixative solution for 10 min, and incubated. The number of positively stained (blue) cells was quantified using Leica microscope imaging.

### 2.17 Cell Culture

The primary human aortic SMCs (HASMC) were obtained from OTWO (Cat number HTX3508, China) and cultivated in Dulbecco's Modified Eagle Medium (DMEM, Cat number SH30243.01, Cytiva) supplemented with 20% fetal bovine serum (FBS) and 1% penicillin/streptomycin. For the cells, a 5% CO_2_ atmosphere was maintained at a constant temperature of 37°C. The cells from passages 4 to 6 were rinsed, subjected to trypsinization, counted, and plated before treatment. Cells were serum‐starved overnight for synchronization.

### 2.18 Cell Transfection and Transduction

Small interfering RNAs (siRNAs) targeting SPSB1 were synthesized by MCE Corporation (Guangzhou, China). Three siRNAs (10 nM) were transfected into cells through RNAiPro Transfection Reagent (Cat number MK4018, MIKX, China) according to the manufacturer's protocol. Non‐targeting siRNAs served as controls. After 24 h, cells were used for subsequent experiments. Table S3 contains the sequences of siRNA.

### 2.19 DIA Proteomics

Aortic SMCs that had been transfected with SPSB1 siRNA were harvested (*n* = 3/group) and subsequently sent to Lianchuan Biotechnology Co. Ltd (Hangzhou, China) for protein extraction, trypsin digestion, data‐independent acquisition (DIA)–mass spectrometry (MS), and data analysis in accordance with established protocols. Differently expressed proteins were filtered using criteria of a fold change of at least 0.263 and a *p*‐value below 0.05. Hierarchical clustering was then conducted. Protein–protein interaction functions were assessed using Gene Ontology (GO) and KEGG pathway analyses.

### 2.20 Enzyme‐Linked Immunosorbent Assay

Using competitive ELISA Kits (Catalog No. EH0201, FineTest, China), we measured the IL‐6 concentrations in the supernatants from cell cultures.

A diluted supernatant aliquot of 100 μL was mixed with 100 μL of the capture and detector antibody cocktail in a 96‐well plate and incubated at room temperature for 1 h. After washing the plate, it was incubated with 90 μL of TMB solution in the dark for 10 min, and absorbance was measured at 450 nm after stopping the reaction.

### 2.21 Gelatin Zymography

The gelatin zymography was executed with the aid of an MMP Zymography assay kit (Cat number P1700, APPLYGEN, China). Equal protein amounts derived from aortic SMCs were run on SDS‐PAGE gels with 1 mg/mL gelatin, incubated in buffer at 37°C overnight, and stained with Coomassie brilliant blue.

### 2.22 Immunofluorescence Staining

In mouse aortic tissue, immunofluorescence was employed to observe α‐SMA (ACTA2) and KLF4. In 96‐well plates, aortic SMCs were plated. The therapies were followed by three washes with PBS. Next, a 4% paraformaldehyde solution was used to stabilize the sample for 15 min. The aortic SMCs were subsequently extracted from the paraformaldehyde and rinsed three times in PBS. The cells were incubated with 5% BSA for 30 min after being permeabilized with 0.1% Triton‐X and rinsed three additional times with PBS. The cells were subsequently incubated with (Cat number 19245s, CST, USA) and KLF4 (Cat number WL02532, Wanlelbio, China) overnight at 4°C, Alexa Fluor 488–labeled secondary antibodies (Cat number P0176, Beyotime, China) overnight at room temperature, and DAPI (Cat number C1005, Beyotime, China) for 5 min at room temperature. Three rinses with PBS were performed on the cells subsequently. A fluorescent microscope (Leica, Germany) was implemented for imaging purposes.

### 2.23 Bulk RNA Sequencing

Total RNA of SPSB1‐silenced aortic SMCs was extracted (*n* = 3/group) mentioned before. Samples were taken to Lianchuan Biotechnology Co. Ltd (Hangzhou, China) for RNA sequencing according to standard protocols. The bulk RNA sequencing (bulk RNA‐seq) data of β‐aminopropionitrile (BAPN)‐treated mice (*n* = 3) and normal mice (*n* = 3) were available from GEO under accession number GSE247088. In the BAPN group, mice were fed with BAPN dissolved in water at a concentration of 0.25% for 4 weeks [23].

The quality of raw reads was initially evaluated using FastQC (Version 0.11.9) software (https://www.bioinformatics.babraham.ac.uk/projects/fastqc/). To obtain clean reads, the raw reads were processed using fastp (Version 0.20.1) software to trim and filter out adapters and reads with low‐quality scores [26].

The clean reads were aligned to the reference genome with HISAT2 software (Version 2.2.1) in the next step [27]. Raw gene counts were estimated by featureCounts (Version 1.6.0) [28]. Differential gene counts of candidate TAA‐related genes were estimated with a *t*‐test between the BAPN‐treated and normal groups. The identification of gene functions was achieved through GO and KEGG pathway analyses.

We employed multivariate analysis of transcript splicing (rMATS) (Version 1.19.2) to undertake AS analysis [29]. rMATS employs default unpaired procedures. In order to detect significant differential splicing events, we established the following cut‐offs: | percent spliced‐in (PSI) | ≥ 0.1 and false discovery rate (FDR) < 0.05.

### 2.24 Statistics

The results for all experiments are expressed as mean ± SD (deviation of the mean). To evaluate the normality of the data, the Shapiro–Wilk test was performed. If the data were normally distributed, a two‐tailed Student's *t*‐test was conducted for two‐group comparisons. Otherwise, the nonparametric Wilcoxon rank sum test was utilized for non‐normally distributed data. To assess the putative correlation of SPSB1 with other genes, a linear regression model was applied. The correction for multiple testing was applied in the corresponding steps. Without the application of multiple testing adjustments, a P‐value of less than 0.05 was viewed as statistically significant. The figure legends provide a comprehensive explanation of the methodologies used for each analysis. R (Version 4.3.1) or GraphPad Prism 9 were employed to conduct all statistical analyses.

## 3 Results

### 3.1 Identification of Traits Relevant to TAA Using Linkage Disequilibrium Score Regression

**FIGURE 1:**
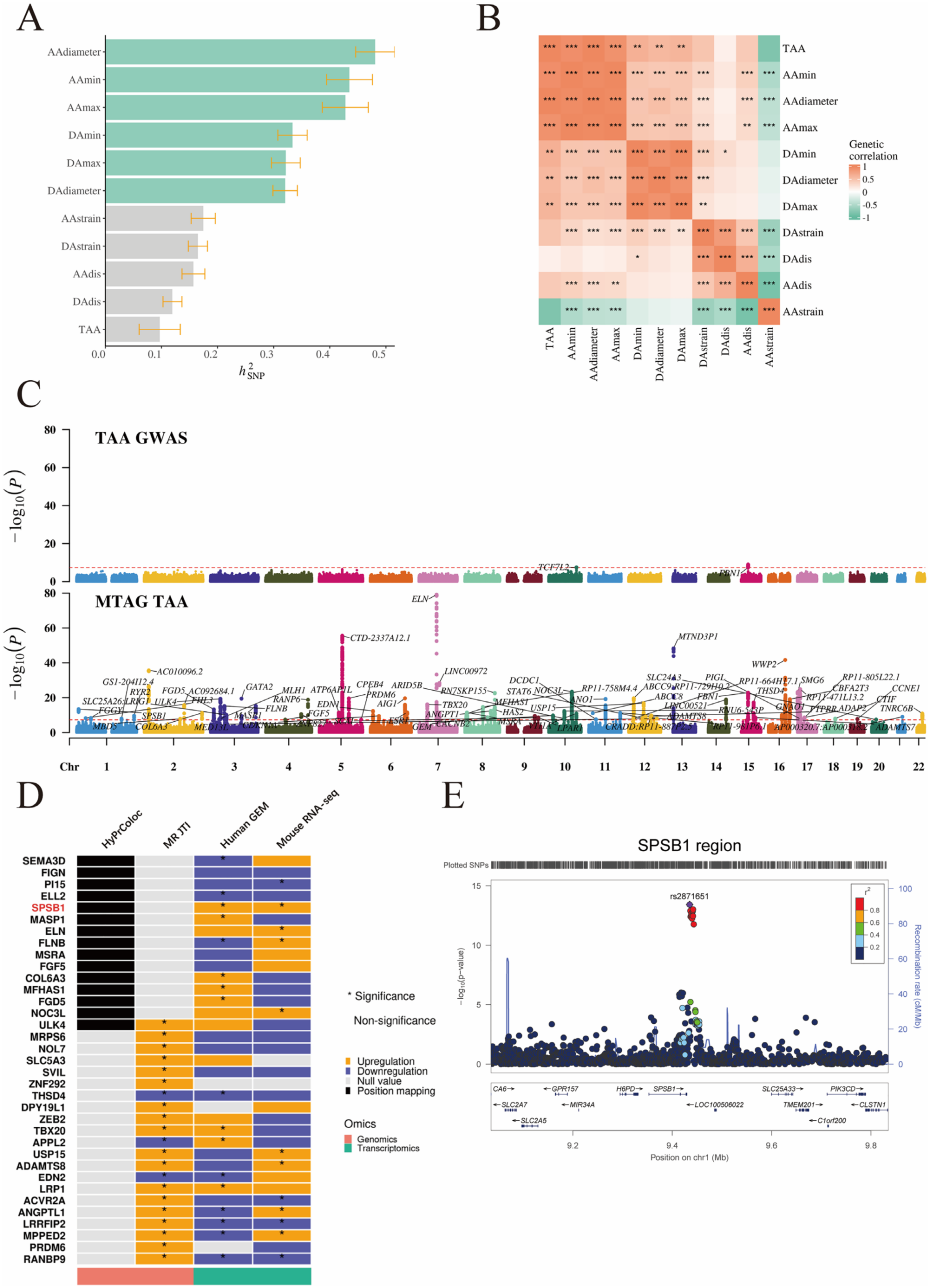
Multi‐traits meta genome‐wide association studies and gene prioritization. (A) Single nucleotide polymorphism (SNP) heritability of thoracic aortic aneurysm (TAA) and thoracic aortic‐related traits; (B) Genetic correlation was shown between thoracic aortic aneurysm (TAA) and other thoracic aortic‐related traits in Linkage disequilibrium score regression (LDSC) analysis. The significance threshold of *P* value was corrected with Bonferroni method. *p < 9.09 × 10^−4^ (0.05/55), **p < 1.82 × 10^−4^ (0.01/55), ***p < 1.82 × 10^−5^ (0.001/55); (C) Manhattan plots of TAA genome‐wide association study (GWAS) and multi‐trait analysis of GWAS (MTAG) TAA. The x‐axis denoted the chromosomal position, and the y‐axis showed the −log_10_ *P*. The horizontal red line corresponded to the genome‐wide significance threshold (*p* < 5 × 10^−8^). Labels were the chromosome regions where genomic risk loci were located. Note that the Manhattan plots were plotted at *P* values truncated by 1 × 10^−80^ for better visualization; (D) Gene prioritization with genomics and transcriptomics. HyproColoc and MR‐JTI are two distinct genomic methods that prioritize candidate genes based on the MTAG results of TAA. Transcriptomic data of TAA from both human (GSE26155) and mouse (GSE247088) were used to validate the genomic findings. A gene was considered a robust candidate if it was significantly expressed in both TAA patients and mice in the same direction, and if the genomic results were not violated; (E) Locuszoom of *SPSB1* locus in TAA GWAS from MTAG results. Rs2871651 (*P*_MTAG_ = 3.8 × 10^−14^) was the top SNP in this locus. TAA, thoracic aortic aneurysm; AAdiameter, ascending aortic diameter; DAdiameter, descending aortic diameter; AAmax, ascending aortic max area; DAmax, descending aortic max area; AAmin, ascending aortic minimum area; DAmin, descending aortic minimum area; AAdis, ascending aortic distensibility; DAdis, descending aortic diameter; AAstrain, ascending aortic strain; DAstrain, descending aortic strain; *h* ^2^_SNP_, heritability; GWAS, genome‐wide association study; MTAG, multi‐trait analysis of GWAS; Chr, chromosome; SNP, single nucleotide polymorphism; *P*_R_, the regional prior; *P*_A_, the alignment prior; SMC, smooth muscle cell.

The liability‐scale SNP heritability (*h* ^2^_snp_) estimates for thoracic aortic–related traits were higher than that of thoracic aortic aneurysm (TAA; *h* ^2^_snp_ = 0.0969, SE = 0.0528). These results suggest that using continuous thoracic aortic–related traits as phenotypes may capture a greater proportion of the genetic variance underlying physiological changes in the thoracic aorta (Figure 1A; Table S4).

Furthermore, we observed that the observed‐scale *h* ^2^_snp_ estimates for thoracic aortic diameter and area were higher than those for thoracic aortic distensibility and strain. Pairwise linkage disequilibrium score regression (LDSC) analysis revealed strong positive genome‐wide genetic correlations between TAA and several aortic measurements: AAdiameter (*r*_g_ = 1.0130, SE = 0.1948; *p* = 2.00 × 10^−7^), AAmax (*r*_g_ = 0.9138, SE = 0.1783; *p* = 3.00 × 10^−7^), AAmin (*r*_g_ = 0.9360, SE = 0.1812; *p* = 2.41 × 10^−7^), DAdiameter (*r*_g_ = 0.5801, SE = 0.1500; *p* = 0.0010), DAmax (*r*_g_ = 0.6065, SE = 0.1506; *p* = 5.65 × 10^−5^), and DAmin (*r*_g_ = 0.6523, SE = 0.1538; *p* = 2.22 × 10^−5^) (Figure 1B; Table S5). Taken together, these findings indicate that thoracic aortic diameter and area are the most relevant traits associated with TAA.

### 3.2 MTAG and N‐GWAMA Analysis Discovering TAA‐Associated Loci

We conducted a meta‐analysis of GWAS summary statistics for thoracic aortic aneurysm (TAA) and six related traits—ascending aortic diameter (AAdiameter), ascending aortic maximum and minimum diameters (AAmax, AAmin), and descending aortic diameter (DAdiameter), as well as descending aortic maximum and minimum diameters (DAmax, DAmin)—using Multi‐Trait Analysis of GWAS (MTAG). In total, 6 431 424 SNPs were included in the MTAG meta‐analysis, of which 4727 reached genome‐wide significance (*p* < 5 × 10^−8^) in the MTAG‐TAA results.

The mean chi‐squared (χ^2^) statistic increased from 1.016 in the original TAA GWAS to 1.273 in MTAG‐TAA, and the number of genome‐wide significant loci rose from 2 to 74 (Figure 1C; Table S6). The genomic inflation factor (λ_GC_) for MTAG‐TAA was 1.2. The maximum false discovery rate (maxFDR) was 0.016, indicating minimal inflation and supporting the assumption of trait homogeneity.

Using N‐GWAMA, the estimated effective sample size of the meta‐analysis was 80 447. Among the 4727 significant SNPs, 3091 met the significance threshold in both MTAG‐TAA (*p* < 5 × 10^−8^) and N‐GWAMA (*p* < 5 × 10^−8^). Additionally, 50 of the 74 independent loci identified in the MTAG analysis were validated (Figure S1; Table S7). Notably, lead SNPs at 8 of the 50 loci were located nearest to previously known TAA‐associated genes: *PI15*, *ELN*, *FLNB*, *ULK4*, *FBN1*, *PRDM6*, *THSD4*, and *MASP1*.

### 3.3 Gene Prioritization With Genomics and Transcriptomics

Using HyPrColoc analysis, we found evidence of colocalization across two or more TAA and thoracic aortic‐related traits at 15 of the 50 validated loci (Figure 1D; Table S8). In the MR‐JTI analysis, 21 protein‐coding genes were identified as significantly associated (Figure 1D; Table S9). Notably, ULK4, a previously reported TAA‐related gene [30], was identified in both analyses.

To further validate these findings, we examined the expression of candidate genes in a gene expression microarray dataset from TAA patients and in bulk RNA‐seq data from BAPN‐induced TAA mice. Statistical significance was determined using the Bonferroni correction (Figure 1D; Table S10). Among the candidates, only SPSB1 reached statistical significance in both human and mouse models, supporting the genomic findings (Figure 1E).

### 3.4 SPSB1 Is Increased in TAA Patients and Murine Models

**FIGURE 2:**
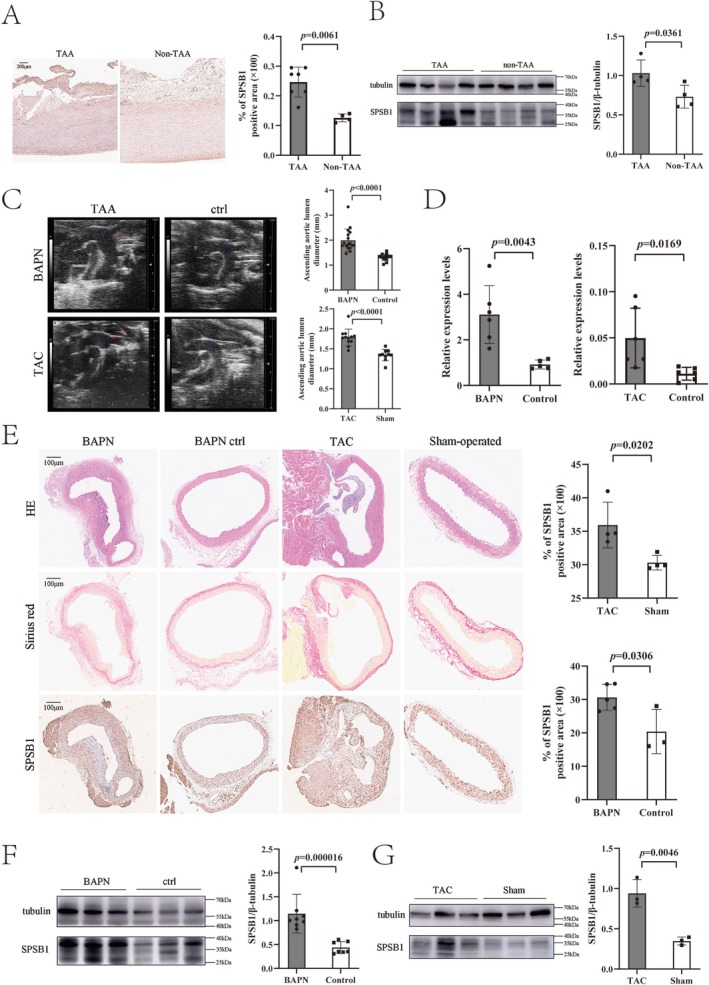
SplA/Ryanodine Receptor Domain and SOCS Box Containing 1 increases in thoracic aortic aneurysm models and thoracic aortic aneurysm patients. (A) Immunostaining for SplA/Ryanodine Receptor Domain and SOCS Box Containing 1 (SPSB1) in thoracic aortic aneurysm (TAA) patients (*n* = 7 in TAA group, *n* = in non‐TAA group). The bar plot show quantification of the percentage of SPSB1 positive areas; (B) Western blot analysis for SPSB1 in TAA patients, β‐tubulin was used as loading control (*n* = 4/groups); (C) Ultrasonic plot of β‐aminopropionitrile (BAPN)‐induced thoracic aortic pathology models, transverse aortic constriction (TAC) models, and corresponding control groups in the phase of end diastole. The bar plot shows the mean lumen diameter of ascending aorta of each group (*n* = 17 in BAPN group, *n* = 15 in BAPN‐control group, *n* = 16 in TAC group, *n* = 15 in TAC sham‐operated group). (D) Real‐time quantitative polymerase chain reaction (qPCR) was used to determine the expression of SPSB1 in BAPN‐induced thoracic aortic pathology models, TAC models, and corresponding control groups (*n* = 6 in BAPN group, *n* = 5 in BAPN‐control group, *n* = 6 in TAC group, *n* = 6 in TAC sham‐operated group). (E) Hematoxylin and eosin staining (H&E), Sirius Red staining, and Immunostaining in the cross‐sections of thoracic aorta (*n* = 5 in BAPN group, *n* = 3 in BAPN‐control group, *n* = 4 in TAC group, *n* = 4 in TAC sham‐operated group). Immunostaining for SPSB1 was conducted in BAPN‐induced thoracic aortic pathology models, TAC models, and corresponding control groups. The bar plot shows quantification of the percentage of SPSB1 positive areas. Statistical analysis was performed using two‐tailed Student's *t*‐test. *p < 0.05; **p < 0.01; ***p < 0.001; (F) Western blot analysis for SPSB1 in BAPN models and control group, β‐tubulin was used as loading control (*n* = 8 in BAPN group, *n* = 7 in control group); (G) Western blot analysis for SPSB1 in TAC models and control group, β‐tubulin was used as loading control (*n* = 6 in TAC group, *n* = 6 in sham‐operated group. In the same group of mice, tissue were randomly pooled in pairs for protein extraction). SiSPSB1, SPSB1 knockdown with small interfering RNAs; ctrl, control group; sham, sham‐operated group.

SPSB1 expression in ascending aortic tissue was assessed through immunostaining and Western blot analyses in 7 TAA patients and 7 non‐TAA control subjects. SPSB1 levels were markedly elevated in TAA patients compared with non‐TAA controls (Figure 2A,B).

To further investigate SPSB1 expression, we analyzed two mouse models of thoracic aortic pathology: the BAPN‐induced model and the transverse aortic constriction (TAC) model, the latter representing a pressure‐overload‐induced ascending aortic aneurysm. In both models, aortic diameters near the right carotid artery increased by 51.7% in BAPN‐induced mice and 32.8% in TAC mice compared with controls (Figure 2C). qPCR confirmed a significant increase in SPSB1 expression in the thoracic aorta of both BAPN‐induced and TAC mice (Figure 2D).

Consistent with findings in human TAA tissue, immunostaining and Western blot analyses demonstrated significantly increased SPSB1 expression in the thoracic aorta of both TAA mouse models compared to control mice. Arterial remodeling appeared in the BAPN and TAC models according to the H&E staining and Sirius Red staining. The results indicated the thoracic aortic pathology models were established successfully (Figure 2E–G).

### 3.5 SPSB1 Regulating SMC Phenotype Switching and Cellular Senescence

**FIGURE 3:**
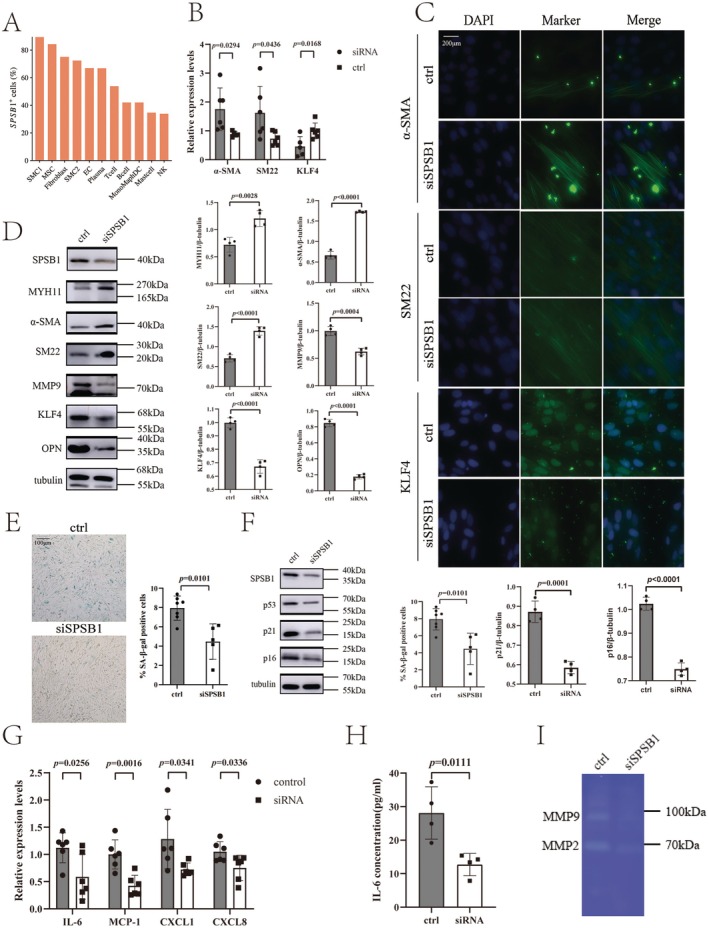
SplA/Ryanodine Receptor Domain and SOCS Box Containing 1 regulates smooth muscle cell phenotype switching and cell senescence. (A) SplA/Ryanodine Receptor Domain and SOCS Box Containing 1 (SPSB1) expression among distinct cellular populations; (B) SPSB1 was knockdown in aortic smooth muscle cell (SMC). The mRNA levels of α‐SMA, SM22, and KLF4 were detected by Real‐time quantitative polymerase chain reaction (qPCR) (*n* = 6/experiments); (C) Immunofluorescent staining for α‐SMA (green), SM22 (green), KLF4 (green), and staining with DAPI (blue) in the aortic SMC after SPSB1 silenced; (D) Western blot analysis of the indicated proteins in SPSB1 knockdown aortic SMCs. β‐tubulin was used as a loading control for Western blotting (*n* = 4/experiments). (E) Representative images of SA‐β‐gal (senescence‐associated β‐galactosidase)–stained SPSB1 knockdown aortic SMC and statistical analysis (right) (*n* = 7 in control group, *n* = 5 in siRNA group). The blue regions are positively stained. Scale bar = 100 μm.; (F) Western blot analysis of senescence markers in SPSB1 knockdown aortic SMCs. β‐tubulin was used as a loading control for Western blotting (*n* = 4/experiments); (G) Quantification of mRNA levels of senescence‐associated secretory phenotype (SASP) components in SPSB1 knockdown aortic SMCs (*n* = 6/experiments). Statistical analysis was performed using two‐tailed Student's *t*‐test; (H) Interleukin 6 (IL‐6) concentration in the culture supernatant of SPSB1 knockdown aortic SMCs. Statistical analysis was performed using two‐tailed Student's *t*‐test (*n* = 4/experiments); (I) The activity of MMP2 and MMP9 in culture medium was measured by gel zymography in SPSB1 knockdown aortic SMCs. SiSPSB1, SPSB1 knockdown with small interfering RNAs; ctrl, control group.

To investigate the distribution of SPSB1 across vascular wall cell lineages, we reanalyzed a single‐cell RNA sequencing (scRNA‐seq) dataset of human aortas (GSE155468). Cell clustering results were visualized using uniform manifold approximation and projection (UMAP) (Figure S2). Among 11 major cell lineages, SPSB1 expression was predominantly enriched in smooth muscle cell subtype 1 (SMC1), with lower levels observed in SMC2 (Figure 3A). SMC1 exhibited higher expression of contractile markers, including *α‐SMA* (*ACTA2*), *MYH11*, and *MYL9*, whereas SMC2 expressed elevated levels of synthetic markers such as *IGFBP2*, *MAP1B*, and *SPARC* (Figure S3). These findings suggest that SPSB1 may play a regulatory role in SMC phenotype switching.

To examine the functional role of SPSB1 in aortic SMC phenotype modulation, we silenced SPSB1 using siRNA. After validating the efficiency of the different siRNA sequences, we selected siRNA Set A‐3 for SPSB1 knockdown (Figure S4). qRT‐PCR analysis revealed increased mRNA expression of contractile markers *SM22* and *α‐SMA*, alongside decreased expression of the synthetic marker *KLF4* (Figure 3B). Immunofluorescence staining demonstrated increased fluorescence intensity of α‐SMA and SM22 but decreased intensity of KLF4 following SPSB1 knockdown (Figure 3C). Consistent with these findings, Western blot analysis showed upregulation of contractile proteins SM22, α‐SMA, and MYH11, along with downregulation of synthetic markers KLF4 and OPN after SPSB1 silencing (Figure 3D).

As aged SMCs are known to accumulate in aortic aneurysms, we further investigated the role of SPSB1 in SMC senescence. Senescence‐associated β‐galactosidase (SA‐β‐gal) staining revealed reduced cellular senescence following SPSB1 knockdown (Figure 3E). Western blot analysis demonstrated decreased expression of senescence markers p53, p21, and p16 (Figure 3F). Additionally, transcriptional analysis confirmed downregulation of senescence‐associated secretory phenotype (SASP) components, including interleukin (IL)‐6 and monocyte chemoattractant protein (MCP)‐1, chemokine (C‐X‐C motif) ligand (CXCL)‐1, and CXCL‐8 (Figure 3G). ELISA showed significantly reduced IL‐6 protein levels in the supernatant of SPSB1‐deficient aortic SMCs (Figure 3H). Gelatin zymography revealed decreased matrix metalloproteinase (MMP) activity following SPSB1 silencing (Figure 3I).

In summary, SPSB1 deficiency inhibits the transition of aortic SMCs from a contractile to a synthetic phenotype and attenuates cellular senescence.

### 3.6 SPSB1 Regulating Global Alternative Pre‐mRNA Splicing Profiles in Aortic SMC

**FIGURE 4:**
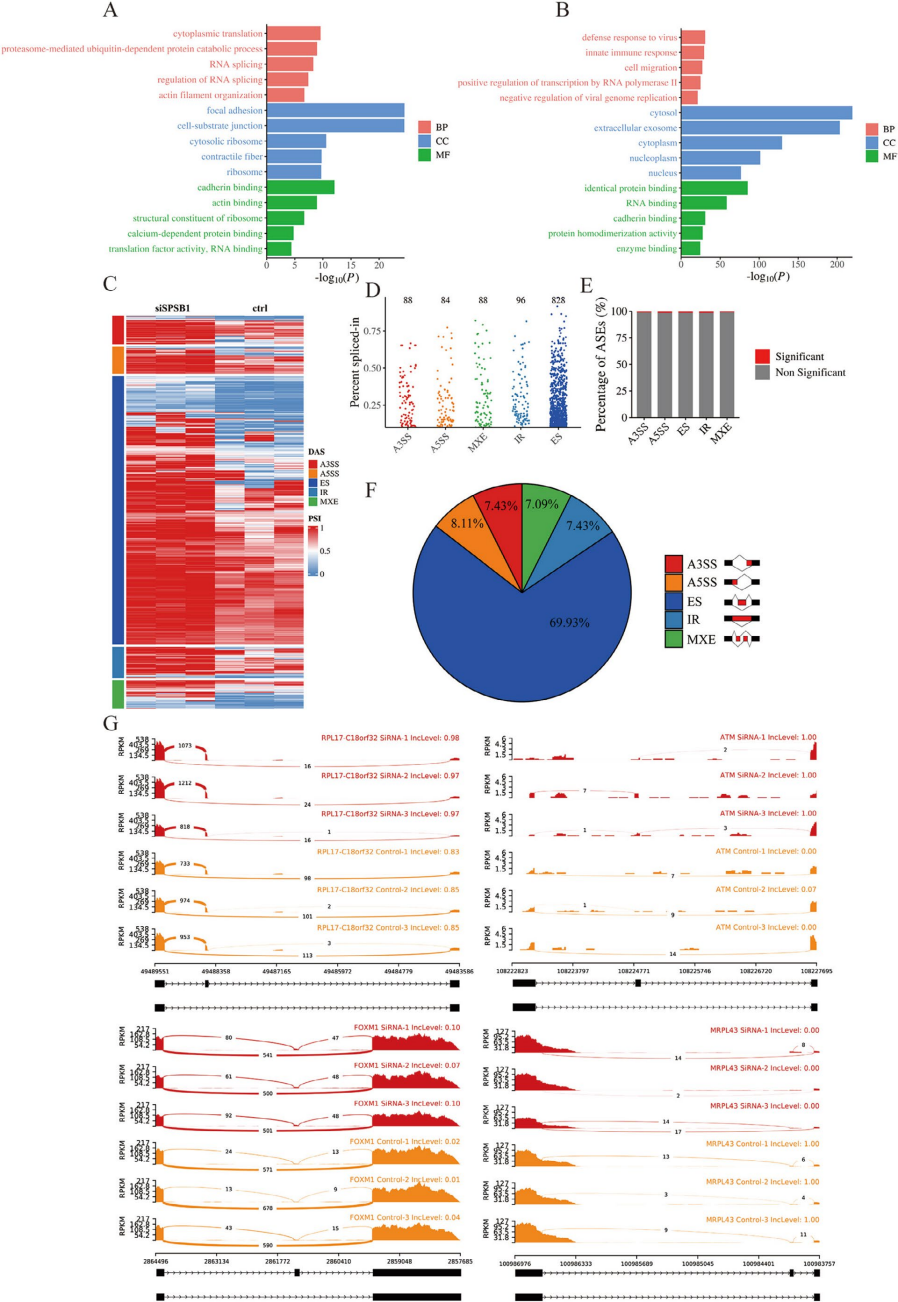
SplA/Ryanodine Receptor Domain and SOCS Box containing 1 regulates global alternative pre‐mRNA splicing profiles in aortic smooth muscle cells. (A) Gene Ontology (GO) enrichment analysis of SplA/Ryanodine Receptor Domain and SOCS Box Containing 1 (SPSB1) co‐expressed genes in contractile smooth muscle cells (SMC) in single‐cell RNA‐sequencing; (B) GO enrichment analysis of quantitative proteomics in SPSB1 knockdown aortic SMCs; (C) Heatmap showing percent spliced‐in (PSI) values for differentially spliced alternative splicing events (DAS) between SPSB1 knockdown aortic SMCs and control group (*n* = 3/group); (D) Dot plot showing the distribution of PSI values for five AS types upon SPSB1 knockdown in aortic SMCs; (E) Bar plot of the DAS observed in RNA‐seq analysis of SPSB1 knockdown aortic SMCs and control group; (F) Schematic (right) and distribution (left) of five alternative splicing event types that differentially change upon SPSB1 knockdown aortic SMCs; (G) Representative Sashimi plot showing the decreased SEs of the RPL17, ATM, FOXM1, and MRPL43 genes in SPSB1 knockdown aortic SMCs and control group. SiSPSB1, SPSB1 knockdown with small interfering RNAs; ctrl, control group; SE, skipped exon; RI, retained intron; A3SS, alternative 3′ splice site; A5SS, alternative 5′ splice site; MXE, mutually exclusive exon.

To elucidate the molecular mechanisms underlying SPSB1 function in aortic SMCs, we re‐analyzed scRNA‐seq data from human aortas. Co‐expression analysis of SPSB1 in the SMC1 population identified 1055 genes with a Bonferroni‐corrected *p* value < 7.1 × 10^−6^ (0.05/7042). Gene Ontology (GO) enrichment analysis of these co‐expressed genes revealed significant enrichment in RNA metabolic processes, including RNA splicing (GO:0008380), regulation of RNA splicing (GO:0043484), and RNA binding (GO:0019843) (Figure 4A; Table S11).

To further investigate the role of SPSB1, we silenced its expression in primary aortic SMCs using siRNA and conducted data‐independent acquisition (DIA)‐based quantitative proteomic analysis. Using *p* < 0.05 and a log_2_ fold‐change threshold of ±0.263, we identified 838 differentially expressed proteins—156 upregulated and 682 downregulated (Figure S5A,B). Enrichment and GO analysis of these proteins indicated a strong association with RNA binding (GO:0019843) (Figure 4B; Table S12). Kyoto encyclopedia of genes and genomes pathway (KEGG) analysis further supported SPSB1's involvement in RNA processing (Figure S5C; Table S12).

To directly assess the role of SPSB1 in alternative splicing (AS), we performed transcriptome profiling of SPSB1‐knockdown and control aortic SMCs using RNA‐seq, followed by percent‐spliced‐in (PSI) analysis with rMATS. SPSB1 knockdown resulted in substantial alterations in AS profiles (Figure 4C). rMATS identified 1184 differential alternative splicing events (AESs) across 873 genes in SPSB1‐deficient SMCs (FDR < 0.05, |ΔPSI| ≥ 0.1) (Figure 4D,E). The most frequent differential ASEs (DAS) were skipped exons (SEs; 69.93%), followed by retained introns (RIs; 7.43%), alternative 3′ splice sites (A3SSs; 7.43%), alternative 5′ splice sites (A5SSs; 8.11%), and mutually exclusive exons (MXEs; 7.09%) (Figure 4F). Representative SE events in genes such as *RPL17*, *ATM*, *FOXM1*, and *MRPL43* were visualized using Sashimi plots (Figure 4G).

Additionally, differential gene expression analysis identified 1068 differentially expressed genes (DEGs) (*q* < 0.05, fold change ≥ 1), including 262 upregulated and 806 downregulated genes in SPSB1‐silenced SMCs compared to controls (Figure S6). Notably, 873 (81.74%) of these DEGs also exhibited DAS. GO and KEGG analyses revealed that SPSB1‐regulated DEGs were significantly associated with the cell cycle (GO:0007049; hsa04110) and cell differentiation (GO:0030154) (Figure S7; Table S13).

Collectively, these results suggest that SPSB1 regulates global alternative splicing and gene expression, thereby influencing phenotypic switching and senescence in aortic SMCs.

## 4 Discussion

We employed a comprehensive study about genetic correlation between TAA and TAA‐related thoracic aortic traits and revealed a strong relationship between TAA, thoracic aortic diameter, and thoracic aortic area. By combining the genomics and transcriptomics data, we discovered SPSB1 is a robust therapeutic target. In vivo, we found SPSB1 increased in TAA mouse models and patients. In vitro, we revealed SPSB1 may be associated with SMC phenotypic switching and cell senescence through alternative splicing, which could help with future disease mechanisms and drug development research.

Diameter‐based measurement was the gold standard to diagnosing TAA and assessing the risk levels [3]. A diameter of aorta ≥ 4.5 cm is defined as TAA due to a 6000‐fold increased risk of dissection in this threshold [31]. However, it is essential to pay attention to a dissection that occurs in a small‐diameter aorta, particularly when the diameter is less than 5.5 cm [29]. In addition, the dissection that occurs at the aortic root may have a lower threshold compared to the supra‐coronary ascending aorta. This indicates a variation in the threshold of risk diameters related to the location of the aorta [32]. The heterogenicity in risk diameter for dissection makes it difficult to determine a reliable threshold for identifying TAA and TAA‐related risk factors. High accuracy and reproducibility in observing the thoracic aorta are achievable due to advancements in both 3D reconstruction technology and image processing tools. This allows for the measurement of the reconstructed 3D geometry rather than 2D images, which may indicate additional TAA indicators [33]. Pirruccello et al. [10] and Francis et al. [11] use deep learning‐based image interpretation methods to measure both ascending and descending aortic diameters and areas through the cardiac cycle. Both thoracic aortic traits showed a strongly genetic correlation with TAA in our study, indicating an important role of diameter‐related traits in TAA progression. In addition, the genetic evidences showed a positive association of aortic diameter and area with hypertension and diastolic blood pressure [10, 11], which were known as the risk factors of TAA [3]. These results suggested a genetic pleiotropy between TAA, thoracic‐aortic traits, and blood pressure‐related traits. Patients with hypertension should focus on the occurrence of TAA. In another research, Pirruccello et al. presented two dynamic‐related traits, strain and distensibility, as measurements to understand the thoracic aortic architecture [12]. Aortic strain and distensibility were the phenotypes related to vascular smooth muscle cell function and extracellular matrix and played a role in several vascular diseases, such as aortopathy, hypertension, and stroke [34]. However, no genetic correlation was observed between TAA and both traits in our study. And it should also be noted that the heritability of aortic distensibility and strain was lower than that of aortic diameter and area, which indicated TAA hereditary effect may be more explained by diam‐related traits rather than dynamic‐related traits. Currently, the GWAS of other aortic diameter‐related traits, such as aortic volume [35], aortic height index [36], and aortic size index is lacking [33]. Our study did not analyze these aortic diameter‐related traits, which could be used as phenotypes to explore the regulatory mechanism of TAA in further research.

We used cross‐trait GWAS meta‐analyses to identify the shared candidate genetic architecture of TAA, thoracic aortic diameter, and thoracic aortic area. We improved the power of gene prioritization by SNP‐level and gene‐level analysis. Thirty five protein‐coding genes were identified as candidate causal genes of TAA. Nine of the 35 genes (including *PI15*, *ELN*, *FLNB*, *ULK4*, *THSD4*, *ZEB2*, *TBX20*, *LRP1*, *PRDM6*) showed an association with TAA in previous studies [5]. It should be noted that both the SNP‐level analysis and gene‐level analysis converged on the same risk gene *ULK4*. In the previous GWAS of acute aortic dissection, rs2272007 was shown as a nonsynonymous SNP in the exonic region of *ULK4* (*P*_MTAG_ = 6.58 × 10^−6^) [30]. In addition, the deletions of *ULK4* were shown as an independent risk factor contributing to the pathogenesis [30]. In our study, rs2272007 (*P*_MTAG_ = 4.2 × 10^−18^, *P*_N‐GWAMA_ = 1.05 × 10^−19^) also showed a significant association with TAA in the *ULK4* region, which further supports that genetic variations in *ULK4* contribute to the pathogenesis of aortic disease. It is noteworthy that the regulatory mechanisms that underlie TAA appear to be more intricate and distinct in the locus 10:95892659–97039458. The lead SNP rs71482305 was responsible for mapping the coding gene NOC3L, which is situated in the intronic region of NOC3L. However, the candidate SNP in this locus, rs10882399, was found to be in proximity to the gene PLCE1, which overlaps with the 3′UTR of NOC3L and has been implicated in the development of aortic aneurysm and dissection [37]. Colocalization analysis also revealed this locus as a causal SNP influencing DAdiameter, DAmax, and DAmin. This suggests that the locus may play distinct roles in the pathogenesis of thoracic aortic aneurysm and descending aortic‐related characteristics.

The vessel wall is primarily composed of SMCs. The formation of TAA is significantly influenced by the diverse alterations in SMC [38]. Phenotypic switching was the most well‐studied change. Stimulation of SMC with cytokines, such as platelet‐derived growth factor‐BB (PDGF‐BB) and transforming growth factor beta (TGF‐β), could upregulate the mRNA and protein expression of contractile proteins (i.e., α‐SMA, SMMHC, and CNN) while reducing proliferation [39]. Some studies suggested cellular senescence of SMC plays a role in TAA formation, which means an upregulation of different cell cycle inhibitors and accumulation of SASP in the SMC [37, 40]. Through integration of large‐scale genomics, transcriptomics, and in vivo experiment validation, we revealed SPSB1 increases in TAA and specifically expressed in SMC. In addition, we found that SPSB1 deficiency inhibited SMC phenotypic transformation and senescence.

SPSB1 is a cullin‐5 E3 ubiquitin ligase adaptor and destabilizes TβRII by enhancing ubiquitination [41]. It recruits Elongin B/C‐Cullin complexes to conjugate lysine 29‐linked polyUb chains onto HNRNPA1, which is a protein essential for alternative splicing [42]. With enrichment analysis of scRNA‐seq and DIA‐MS, we revealed that SPSB1 was related to alternative splicing. RNA‐seq of aortic SMC after SPSB1 silencing revealed a global change of ASEs. Therefore, we suggested that SPSB1 is involved in alternative splicing in the SMC of TAA, which may be related to HNRNPA1.

Taken together, we identified thoracic aortic diameter and area with significant genetic correlation with TAA. By integrating multi‐omics data and in vivo experiments, we discovered SPSB1 related to TAA. Further experiments showed that SPSB1 may play a role in SMC phenotype switching and cell senescence through alternative splicing. These discoveries will offer novel insights into the fundamental biology and will facilitate the exploration of potential therapeutics in TAA.

## 5 Study Limitations

Our study has several limitations. First, for genetic correlation and cross‐trait meta‐analysis, we mainly used processed GWAS summary statistics from the United Kingdom Biobank. However, we did not have another independent dataset as cross‐validation for our observations. Furthermore, we did not manually select the phenotype to avoid potential selection bias. This limitation highlights the need for future studies using independent datasets to confirm the biological relevance of the identified SNPs and candidate genes in TAA pathogenesis. Second, although our results suggested that SPSB1 was the most robust risk gene in TAA through multi‐omics integration analysis, the population of samples in the GWAS, transcriptomics, and in vivo experiments was different. In addition, due to the lack of individual‐level data from the United Kingdom Biobank, we are unable to perform sex‐stratified GWAS analysis specifically for SPSB1. In the future, additional studies are needed to investigate whether population differences may affect the robustness of the candidate genes related to TAA. Third, although our study suggests a potential association between SPSB1 and SMC phenotype switching, as well as cell senescence through alternative splicing, causality cannot be conclusively established based solely on the elevated levels of SPSB1 observed in TAA tissues. Furthermore, our study did not fully address the functional consequences of the identified SNPs on gene expression. Additionally, since SPSB1 is not a spliceosome component, the proposed alternative splicing events resulting from SPSB1 knockdown require further experimental validation to establish a direct link to SMC phenotypic switching. Further studies are needed to provide more evidence to explore the mechanism of SPSB1 in TAA.

## 6 Importance Statement

This study was the first to use genome‐wide association studies (GWAS) data of TAA and other thoracic aortic related traits for studying shared genetic architecture and discovered the novel potential targets of TAA. We integrated GWAS, bulk RNA sequence, single‐cell RNA sequence, and quantitative proteomic data to improve the robustness of the findings. We demonstrated a high expression level of SPSB1 and an elevated risk of TAA. This conclusion was corroborated by BAPN‐induced animal models, TAC animal models, and TAA patients. We suggested SPSB1 might serve an effect in smooth muscle cell phenotypic switching and cell senescence through alternative splicing, providing new insight into the TAA mechanism.

## Author Contributions

Qingyang Song, Tongxin Chu, and Quan Liu participated in conceiving and designing the study, reviewing, performing statistical analysis, and drafting the manuscript. Rennan Weng participated in drafting the manuscript. Huayang Li and Mengya Liang participated in conceiving and designing the study, interpreting data, and revising the manuscript critically and intensively for the intellectual content. Zhongkai Wu conceived and participated in the study design, reviewed and edited the manuscript, had full access to all the data in the study, and took responsibility for the integrity of the data and the accuracy of the data analysis. All authors approved the final manuscript.

## Ethics Statement

All animal experiments were performed in compliance with the guidelines for the care and use of laboratory animals and were approved by the ethics committee of the China Sun Yat‐sen University (SYSU‐IACUC‐2024‐001388). The protocol for collecting human aortic tissue samples was approved by the Ethics Committee of the First Affiliated Hospital of Sun Yat‐Sen University ([2018]118). All experiments involving human aortic tissue samples were performed in accordance with the guidelines approved by the committee. Informed consent was obtained from all participants or donor/recipient families.

## Consent

The content of the manuscript has not been published, or submitted for publication elsewhere, and all authors have approved the manuscript for submission.

## Conflicts of Interest

The authors declare no conflicts of interest.

## Supporting information


**Table S1:** fsb271117‐sup‐0001‐TableS1.docx.

**Table S2:** fsb271117‐sup‐0002‐TableS2.docx.

**Table S3:** fsb271117‐sup‐0003‐TableS3.docx.

**Table S4:** fsb271117‐sup‐0004‐TableS4.docx.

**Table S5:** fsb271117‐sup‐0005‐TableS5.docx.

**Table S6:** fsb271117‐sup‐0006‐TableS6.docx.

**Table S7:** fsb271117‐sup‐0007‐TableS7.docx.

**Table S8:** fsb271117‐sup‐0008‐TableS8.docx.

**Table S9:** fsb271117‐sup‐0009‐TableS9.docx.

**Table S10:** fsb271117‐sup‐0010‐TableS10.docx.

**Table S11:** fsb271117‐sup‐0011‐TableS11.docx.

**Table S12:** fsb271117‐sup‐0012‐TableS12.docx.

**Table S13:** fsb271117‐sup‐0013‐TableS13.docx.

**Figure S1:** fsb271117‐sup‐0014‐FigureS1.tif.

**Figure S2:** fsb271117‐sup‐0015‐FigureS2.tif.

**Figure S3:** fsb271117‐sup‐0016‐FigureS3.tif.

**Figure S4:** fsb271117‐sup‐0017‐FigureS4.tif.

**Figure S5:** fsb271117‐sup‐0018‐FigureS5.tif.

**Figure S6:** fsb271117‐sup‐0019‐FigureS6.tif.

**Figure S7:** fsb271117‐sup‐0020‐FigureS7.tif.

## Data Availability

The datasets supporting the conclusions of this article are included within the article (and its additional file(s)).
