# Supplementary material for: Dissecting Shared Genetic Architecture of Thoracic Aortic Aneurysm and Aortic Related Traits and Identifying SplA/Ryanodine Receptor Domain and SOCS Box Containing 1 Involved in Smooth Muscle Phenotype Switching and Cell Senescence Through Alternative Splicing
Source: FASEB J. 2025 Nov 18;39(22):e71117. doi: 10.1096/fj.202502457R (PMC12637301; doi:10.1096/fj.202502457R)
Supplement: Supplementary file 1 — Table S1: fsb271117‐sup‐0001‐TableS1.docx. [file FSB2-39-e71117-s020.docx]

**Supplemental Table S1. Information of the used Genome-wide association studies**

| **Phenotype** | **Data source** | **Population** | **Individuals** | **Reference genome** | **URL** |
| --- | --- | --- | --- | --- | --- |
| Thoracic aortic aneurysm | UKbiobank | European | 1351 cases 18295 controls | GRCh37 (hg19) | <https://www.ebi.ac.uk/gwas/studies/GCST90027266> |
| Thoracic aortic diameter | UKbiobank | European | 39688 individuals | GRCh37 (hg19) | <https://www.ebi.ac.uk/gwas/studies/GCST90094400> |
| Thoracic aortic distensibility and strain | UKbiobank | European | 42342 individuals | GRCh37 (hg19) | <https://www.ebi.ac.uk/gwas/publications/37019578> |
| Thoracic aortic area | UKbiobank | European | 32590 individuals | GRCh37 (hg19) | <https://data.hpc.imperial.ac.uk/resolve/?doi=10654&access=> |
