# Supplementary material for: Dissecting Shared Genetic Architecture of Thoracic Aortic Aneurysm and Aortic Related Traits and Identifying SplA/Ryanodine Receptor Domain and SOCS Box Containing 1 Involved in Smooth Muscle Phenotype Switching and Cell Senescence Through Alternative Splicing
Source: FASEB J. 2025 Nov 18;39(22):e71117. doi: 10.1096/fj.202502457R (PMC12637301; doi:10.1096/fj.202502457R)
Supplement: Supplementary file 2 — Table S2: fsb271117‐sup‐0002‐TableS2.docx. [file FSB2-39-e71117-s012.docx]

**Supplemental Table S2. Characteristics of the enrolled patients**

| **Variable** | **TAA** | **Control** |
| --- | --- | --- |
| N | 7 | 7 |
| Sex |  |  |
| Female | 1 (14.3 %) | 1 (14.3 %) |
| Male | 6 (85.7 %) | 6 (85.7 %) |
| Age (years) | 54.1 (8.4) | 41.4 (24.0) |
| Height (cm) | 164.1 (4.0) | 167.1 (6.2) |
| Weight (kg) | 54.3 (2.8) | 56.4 (10.8) |
| BMI (kg/m^2) | 20.1 (0.1) | 20.0 2.7) |
| Systolic pressure (mmHg) | 148 (17) | 122 (10) |
| Diastolic pressure (mmHg) | 80 (20) | 70 (10) |
| Aortic diameter (cm) | 5.93 (1.56) | 2.76 (0.59) |
| Smoking status | 2 (28.6%) | 1 (14.3 %) |
| Diabetes mellitus | 0 (0) | 2 (28.6%) |
| Hypertension | 6 (85.7%) | 3 (42.9%) |
| Reoperation | 0 (0) | 0 (0) |
