# Supplementary material for: Dissecting Shared Genetic Architecture of Thoracic Aortic Aneurysm and Aortic Related Traits and Identifying SplA/Ryanodine Receptor Domain and SOCS Box Containing 1 Involved in Smooth Muscle Phenotype Switching and Cell Senescence Through Alternative Splicing
Source: FASEB J. 2025 Nov 18;39(22):e71117. doi: 10.1096/fj.202502457R (PMC12637301; doi:10.1096/fj.202502457R)
Supplement: Supplementary file 3 — Table S3: fsb271117‐sup‐0003‐TableS3.docx. [file FSB2-39-e71117-s008.docx]

| **Supplemental Table S3. Information of real-time PCR primer** **sequences and small interfering RNAs sequences** | | | |  |  |  |
| --- | --- | --- | --- | --- | --- | --- |
| \| **Gene** \| **Forward primer** \| **Reverse primer** \| \| --- \| --- \| --- \| \| **Mouse** \|  \|  \| \| SPSB1 \| CATGCGGGACCCCACATAC \| GCTGCACATCGTAGGACACAG \| \| GAPDH \| GGGTCCCAGCTTAGGTTCATC \| CCAATACGGCCAAATCCGTTC \| \| **Human** \|  \|  \| \| SPSB1 \| AGTACATGGGAGTGGCTTTTC \| ACAAATCCATGAGCGGCAG \| \| GAPDH \| GAACGGGAAGCTCACTGG \| GCCTGCTTCACCACCTTCT \| \| IL-6 \| ACCCCCAGGAGAAGATTCCA \| GCCTCTTTGCTGCTTTCACA \| \| MCP-1 \| CAGCCAGATGCAATCAATGCC \| TGGAATCCTGAACCCACTTCT \| \| CXCL-1 \| ACATCCAAAGTGTGAACGTGA \| ATGGGGGATGCAGGATTGAG \| \| CXCL-8 \| AAGGTGCAGTTTTGCCAAGG \| CCCAGTTTTCCTTGGGGTCC \| | | | |  |  |  |
|  |  |  |  |  |  |  |
|  |  |  |  |  |  |  |

| **Sequences for siRNAs targeting SPSB1** | |
| --- | --- |
| siRNA Set A-1 | GGAUCAAGACUGUGGACAUUU |
|  | AUGUCCACAGUCUUGAUCCUU |
| siRNA Set A-2 | GCAAGAACCAGCCAAGCAAUU |
|  | UUGCUUGGCUGGUUCUUGCUU |
| siRNA Set A-3 | ACACAACCCUCGUGGGGAAUU |
|  | UUCCCCACGAGGGUUGUGUUU |
