# Supplementary material for: Dissecting Shared Genetic Architecture of Thoracic Aortic Aneurysm and Aortic Related Traits and Identifying SplA/Ryanodine Receptor Domain and SOCS Box Containing 1 Involved in Smooth Muscle Phenotype Switching and Cell Senescence Through Alternative Splicing
Source: FASEB J. 2025 Nov 18;39(22):e71117. doi: 10.1096/fj.202502457R (PMC12637301; doi:10.1096/fj.202502457R)
Supplement: Supplementary file 4 — Table S4: fsb271117‐sup‐0004‐TableS4.docx. [file FSB2-39-e71117-s015.docx]

**Supplemental Table S4. Information of SNP heritability of TAA and thoracic aortic-related traits**

| **Phenotype** | **SNP heritability** | **Standard error** |
| --- | --- | --- |
| Thoracic aortic aneurysm | 0.0969 | 0.0366 |
| Descending thoracic aortic distensibility | 0.1196 | 0.0169 |
| Ascending thoracic aortic distensibility | 0.157 | 0.0204 |
| Descending thoracic aortic strain | 0.1649 | 0.017 |
| Ascending thoracic strain | 0.1745 | 0.0215 |
| Descending thoracic aortic diameter | 0.3204 | 0.0218 |
| Descending thoracic aortic max area | 0.3217 | 0.0256 |
| Descending thoracic aortic minimum area | 0.3336 | 0.0261 |
| Ascending thoracic aortic max area | 0.4277 | 0.041 |
| Ascending thoracic aortic minimum area | 0.4351 | 0.0409 |
| Ascending thoracic aortic diameter | 0.4809 | 0.0348 |

SNP, single nucleotide polymorphisms
