# Supplementary material for: Dissecting Shared Genetic Architecture of Thoracic Aortic Aneurysm and Aortic Related Traits and Identifying SplA/Ryanodine Receptor Domain and SOCS Box Containing 1 Involved in Smooth Muscle Phenotype Switching and Cell Senescence Through Alternative Splicing
Source: FASEB J. 2025 Nov 18;39(22):e71117. doi: 10.1096/fj.202502457R (PMC12637301; doi:10.1096/fj.202502457R)
Supplement: Supplementary file 5 — Table S5: fsb271117‐sup‐0005‐TableS5.docx. [file FSB2-39-e71117-s010.docx]

**Supplemental Table S5. Genetic correlation between TAA and all thoracic aortic-related traits**

| **Trait1** | **Trait2** | **Genetic correlation** | **Standard error** | ***P*** |
| --- | --- | --- | --- | --- |
| TAA | AAdiameter | 1.013 | 0.1948 | 2.00E-07 |
| TAA | DAdiameter | 0.5801 | 0.15 | 0.0001 |
| TAA | AAmax | 0.9138 | 0.1783 | 3.00E-07 |
| TAA | AAmin | 0.936 | 0.1812 | 2.41E-07 |
| TAA | DAmax | 0.6065 | 0.1506 | 5.65E-05 |
| TAA | DAmin | 0.6523 | 0.1538 | 2.22E-05 |
| TAA | AAdis | 0.4342 | 0.1638 | 0.008 |
| TAA | AAstrain | -0.8252 | 0.3087 | 7.50E-03 |
| TAA | DAdis | 0.1191 | 0.1818 | 5.13E-01 |
| TAA | DAstrain | 0.4755 | 0.1583 | 0.0027 |
| AAdiameter | DAdiameter | 0.5291 | 0.0362 | 1.91E-48 |
| AAdiameter | AAmax | 0.9771 | 0.0099 | 0 |
| AAdiameter | AAmin | 0.9687 | 0.01 | 0 |
| AAdiameter | DAmax | 0.4554 | 0.0423 | 5.22E-27 |
| AAdiameter | DAmin | 0.4668 | 0.0422 | 1.69E-28 |
| AAdiameter | AAdis | 0.3058 | 0.0646 | 2.18E-06 |
| AAdiameter | AAstrain | -0.4246 | 0.0795 | 9.26E-08 |
| AAdiameter | DAdis | 0.1138 | 0.0762 | 0.135 |
| AAdiameter | DAstrain | 0.2607 | 0.0581 | 7.11E-06 |
| DAdiameter | AAmax | 0.4592 | 0.0453 | 3.74E-24 |
| DAdiameter | AAmin | 0.4468 | 0.045 | 2.79E-23 |
| DAdiameter | DAmax | 0.9137 | 0.0176 | 0.00E+00 |
| DAdiameter | DAmin | 0.9127 | 0.0172 | 0 |
| DAdiameter | AAdis | 0.1596 | 0.0599 | 7.70E-03 |
| DAdiameter | AAstrain | -0.1612 | 0.0689 | 0.0193 |
| DAdiameter | DAdis | 0.204 | 0.0695 | 3.30E-03 |
| DAdiameter | DAstrain | 0.3016 | 0.0574 | 1.51E-07 |
| AAmax | AAmin | 0.9948 | 0.0013 | 0 |
| AAmax | DAmax | 0.4655 | 0.0467 | 2.09E-23 |
| AAmax | DAmin | 0.4754 | 0.046 | 4.72E-25 |
| AAmin | DAmin | 0.4789 | 0.0449 | 1.56E-26 |
| DAmax | AAmin | 0.4587 | 0.0459 | 1.53E-23 |
| DAmax | DAmin | 0.9871 | 0.0022 | 0 |
| AAdis | AAmax | 0.2923 | 0.0734 | 6.88E-05 |
| AAdis | AAmin | 0.3809 | 0.0692 | 3.68E-08 |
| AAdis | DAmax | 0.1358 | 0.0615 | 0.0272 |
| AAdis | DAmin | 0.1945 | 0.0604 | 0.0013 |
| AAdis | AAstrain | -0.8637 | 0.0291 | 3.96E-193 |
| AAdis | DAdis | 0.651 | 0.0623 | 1.52E-25 |
| AAdis | DAstrain | 0.4575 | 0.0757 | 1.48E-09 |
| AAstrain | AAmax | -0.4083 | 0.092 | 9.09E-06 |
| AAstrain | AAmin | -0.5144 | 0.0846 | 1.21E-09 |
| AAstrain | DAmax | -0.1313 | 0.0741 | 0.0765 |
| AAstrain | DAmin | -0.2284 | 0.073 | 0.0018 |
| AAstrain | DAstrain | -0.6361 | 0.068 | 8.60E-21 |
| DAdis | AAmax | 0.1448 | 0.0864 | 0.0937 |
| DAdis | AAmin | 0.1933 | 0.084 | 2.13E-02 |
| DAdis | DAmax | 0.1513 | 0.0755 | 4.50E-02 |
| DAdis | DAmin | 0.2703 | 0.0715 | 2.00E-04 |
| DAdis | AAstrain | -0.5385 | 0.092 | 4.75E-09 |
| DAdis | DAstrain | 0.829 | 0.0329 | 3.23E-140 |
| DAstrain | AAmax | 0.2837 | 0.0621 | 4.97E-06 |
| DAstrain | AAmin | 0.3389 | 0.0601 | 1.71E-08 |
| DAstrain | DAmax | 0.2311 | 0.0601 | 1.00E-04 |
| DAstrain | DAmin | 0.3879 | 0.0549 | 1.59E-12 |

AAdiameter, ascending thoracic aortic diameter; DAdiameter, descending thoracic aortic diameter; AAmax, ascending thoracic aortic max area; AAmin, ascending thoracic aortic minimum area; DAmax, descending thoracic aortic max area; DAmin, descending thoracic aortic minimum area; AAdis, ascending thoracic aortic distensibility; DAdis, descending thoracic aortic distensibility; AAstrian, ascending thoracic strain; DAstrain, descending thoracic aortic strain.
