# Supplementary material for: Dissecting Shared Genetic Architecture of Thoracic Aortic Aneurysm and Aortic Related Traits and Identifying SplA/Ryanodine Receptor Domain and SOCS Box Containing 1 Involved in Smooth Muscle Phenotype Switching and Cell Senescence Through Alternative Splicing
Source: FASEB J. 2025 Nov 18;39(22):e71117. doi: 10.1096/fj.202502457R (PMC12637301; doi:10.1096/fj.202502457R)
Supplement: Supplementary file 6 — Table S6: fsb271117‐sup‐0006‐TableS6.docx. [file FSB2-39-e71117-s004.docx]

**Supplemental Table S6. Information of genomic loci of TAA GWAS and MTAG**

| **Genomic Locus** | **Unique ID** | **rsID** | **chr** | **pos** | ***p*** | **Nearest gene** |
| --- | --- | --- | --- | --- | --- | --- |
| **TAA GWAS** |  |  |  |  |  |  |
| 10:114746580-114748617 | 10:114747277:A:G | rs4073288 | 10 | 114747277 | 3.09E-08 | TCF7L2 |
| 15:48691494-48939888 | 15:48894200:A:T | rs591519 | 15 | 48894200 | 1.06E-09 | FBN1 |
| **MTAG TAA** |  |  |  |  |  |  |
| 1:9434969-9443971 | 1:9434969:C:T | rs2871651 | 1 | 9434969 | 3.80E-14 | SPSB1 |
| 1:38397341-38463504 | 1:38461821:C:T | rs67631072 | 1 | 38461821 | 5.75E-09 | FHL3 |
| 1:59613905-59887078 | 1:59881472:C:T | rs657746 | 1 | 59881472 | 4.35E-09 | FGGY |
| 1:185618884-186031363 | 1:185663021:C:T | rs72727759 | 1 | 1.86E+08 | 1.09E-08 | GS1-204I12.4 |
| 1:237194922-237210003 | 1:237194922:A:G | rs10802580 | 1 | 2.37E+08 | 2.11E-10 | RYR2 |
| 2:19693806-19772505 | 2:19725556:A:G | rs824510 | 2 | 19725556 | 2.01E-36 | AC010096.2 |
| 2:148457312-148915190 | 2:148799710:A:C | rs12992231 | 2 | 1.49E+08 | 1.37E-08 | MBD5 |
| 2:164752160-164930382 | 2:164906820:C:T | rs16849225 | 2 | 1.65E+08 | 1.88E-16 | AC092684.1 |
| 2:238210049-238233483 | 2:238227594:A:G | rs12052878 | 2 | 2.38E+08 | 5.26E-12 | COL6A3 |
| 3:14818716-14928729 | 3:14863636:A:G | rs73028182 | 3 | 14863636 | 5.45E-14 | FGD5 |
| 3:36966449-37326663 | 3:37075934:C:T | rs6772548 | 3 | 37075934 | 2.12E-08 | MLH1 |
| 3:41749669-42159179 | 3:41755359:C:T | rs9847006 | 3 | 41755359 | 4.48E-20 | ULK4 |
| 3:58049639-58199669 | 3:58146711:A:G | rs9817209 | 3 | 58146711 | 5.38E-16 | FLNB |
| 3:66325234-66448307 | 3:66434643:C:T | rs2306272 | 3 | 66434643 | 7.15E-11 | SLC25A26:LRIG1 |
| 3:128198980-128682169 | 3:128201889:C:T | rs62270945 | 3 | 1.28E+08 | 3.50E-20 | GATA2 |
| 3:186947411-187018653 | 3:186997742:G:T | rs698083 | 3 | 1.87E+08 | 4.69E-16 | MASP1 |
| 4:81164723-81202048 | 4:81202048:G:T | rs36034102 | 4 | 81202048 | 3.85E-08 | FGF5 |
| 4:146756490-146821725 | 4:146800815:A:G | rs1979974 | 4 | 1.47E+08 | 2.63E-11 | ZNF827 |
| 4:174656889-174690452 | 4:174656889:A:G | rs67846163 | 4 | 1.75E+08 | 1.55E-19 | RANP6 |
| 5:81716027-81896198 | 5:81723109:C:T | rs2897603 | 5 | 81723109 | 3.37E-12 | ATP6AP1L |
| 5:95162219-95776105 | 5:95582494:A:G | rs4077816 | 5 | 95582494 | 2.58E-56 | CTD-2337A12.1 |
| 5:121942164-122642381 | 5:122531347:A:G | rs17470137 | 5 | 1.23E+08 | 2.51E-20 | PRDM6 |
| 5:173272518-173385908 | 5:173285545:A:G | rs6894235 | 5 | 1.73E+08 | 3.64E-08 | CPEB4 |
| 6:12278956-12587128 | 6:12295987:C:T | rs1630736 | 6 | 12295987 | 4.43E-13 | EDN1 |
| 6:36618821-36649593 | 6:36647289:A:G | rs3176326 | 6 | 36647289 | 3.13E-10 | CDKN1A |
| 6:143437445-143719278 | 6:143592386:A:G | rs1570350 | 6 | 1.44E+08 | 2.29E-20 | AIG1 |
| 6:152308973-152411198 | 6:152333104:A:G | rs13203975 | 6 | 1.52E+08 | 2.62E-12 | ESR1 |
| 7:34941004-35545787 | 7:35282931:A:C | rs10486660 | 7 | 35282931 | 6.66E-17 | TBX20 |
| 7:73293811-73567718 | 7:73428222:A:G | rs6974735 | 7 | 73428222 | 6.84E-80 | ELN |
| 7:84875267-85177938 | 7:85034227:G:T | rs1583081 | 7 | 85034227 | 3.12E-29 | LINC00972 |
| 8:8088230-8922464 | 8:8684953:A:G | rs1533059 | 8 | 8684953 | 1.68E-11 | MFHAS1 |
| 8:9708433-11878338 | 8:10079637:A:G | rs4840467 | 8 | 10079637 | 2.90E-12 | MSRA |
| 8:75540855-75788406 | 8:75774738:A:G | rs2570182 | 8 | 75774738 | 2.25E-15 | RP11-758M4.4 |
| 8:95250558-95281278 | 8:95267597:A:G | rs16916666 | 8 | 95267597 | 1.94E-09 | GEM |
| 8:108286782-108536949 | 8:108294144:C:T | rs7845785 | 8 | 1.08E+08 | 3.97E-13 | ANGPT1 |
| 8:122627847-122702987 | 8:122647015:C:T | rs11780881 | 8 | 1.23E+08 | 4.74E-14 | HAS2 |
| 8:124541280-124615765 | 8:124607159:C:T | rs34557926 | 8 | 1.25E+08 | 1.74E-23 | RN7SKP155 |
| 9:113648136-113776692 | 9:113664990:A:G | rs2182578 | 9 | 1.14E+08 | 2.54E-10 | LPAR1 |
| 9:127766897-127988241 | 9:127820351:A:C | rs11793512 | 9 | 1.28E+08 | 2.57E-09 | SCAI |
| 10:18506911-18535616 | 10:18514999:A:G | rs1757223 | 10 | 18514999 | 1.49E-10 | CACNB2 |
| 10:63805335-63825807 | 10:63818152:C:T | rs7908159 | 10 | 63818152 | 1.05E-08 | ARID5B |
| 10:64874754-65400080 | 10:64876554:A:G | rs7084569 | 10 | 64876554 | 4.67E-13 | RNU6-543P |
| 10:95892659-97039458 | 10:96119130:C:T | rs71482305 | 10 | 96119130 | 3.66E-24 | NOC3L |
| 10:114449904-114520134 | 10:114487697:C:T | rs34943800 | 10 | 1.14E+08 | 1.14E-11 | VTI1A |
| 11:17498057-17498057 | 11:17498057:A:G | rs77889556 | 11 | 17498057 | 7.99E-12 | ABCC8 |
| 11:30798288-31020084 | 11:30851976:C:T | rs3741025 | 11 | 30851976 | 2.67E-08 | DCDC1 |
| 11:69791952-70055721 | 11:70005374:A:C | rs875107 | 11 | 70005374 | 5.15E-20 | ANO1 |
| 11:130266117-130307440 | 11:130271647:A:G | rs747249 | 11 | 1.3E+08 | 5.65E-12 | ADAMTS8 |
| 12:20144754-20249314 | 12:20244030:A:G | rs4237919 | 12 | 20244030 | 9.44E-09 | RP11-664H17.1 |
| 12:21859049-22047455 | 12:22005003:G:T | rs2307024 | 12 | 22005003 | 2.22E-20 | ABCC9:RP11-729I10.2 |
| 12:57502981-57533690 | 12:57514554:C:T | rs10876963 | 12 | 57514554 | 6.08E-09 | STAT6 |
| 12:62571546-62825851 | 12:62763952:C:T | rs56059115 | 12 | 62763952 | 1.43E-17 | USP15 |
| 12:71096471-71114400 | 12:71106648:C:T | rs10879184 | 12 | 71106648 | 5.79E-10 | PTPRR |
| 12:89941549-90091782 | 12:89950320:A:C | rs7302816 | 12 | 89950320 | 2.47E-09 | RP11-981P6.1 |
| 12:94090867-94181470 | 12:94122219:C:T | rs2885691 | 12 | 94122219 | 1.58E-10 | CRADD:RP11-887P2.5 |
| 12:116756670-116756670 | 12:116756670:G:T | rs61937394 | 12 | 1.17E+08 | 1.01E-08 | MED13L |
| 13:22852274-22906527 | 13:22871446:A:G | rs7994761 | 13 | 22871446 | 4.49E-49 | MTND3P1 |
| 14:94423922-94535823 | 14:94459845:A:G | rs4905134 | 14 | 94459845 | 1.23E-19 | LINC00521 |
| 15:48691494-48944563 | 15:48862043:A:G | rs1848050 | 15 | 48862043 | 1.25E-23 | FBN1 |
| 15:71587373-71703643 | 15:71612514:G:T | rs1441358 | 15 | 71612514 | 5.43E-18 | THSD4 |
| 15:78942349-79099145 | 15:79052580:A:G | rs12906653 | 15 | 79052580 | 2.53E-09 | ADAMTS7 |
| 16:55907600-56334990 | 16:56229578:A:G | rs959935 | 16 | 56229578 | 7.50E-11 | GNAO1 |
| 16:66709863-72927031 | 16:69965021:C:T | rs77870048 | 16 | 69965021 | 2.12E-42 | WWP2 |
| 16:88966667-89032631 | 16:88989862:A:G | rs16965180 | 16 | 88989862 | 7.82E-20 | CBFA2T3 |
| 17:1931965-2333758 | 17:2097483:G:T | rs1532292 | 17 | 2097483 | 4.83E-24 | SMG6 |
| 17:12157215-12194059 | 17:12182246:A:G | rs7215383 | 17 | 12182246 | 1.16E-25 | RP11-471L13.2 |
| 17:15870401-16263682 | 17:16139149:C:T | rs12945855 | 17 | 16139149 | 2.70E-08 | PIGL |
| 17:29021580-29263700 | 17:29263700:C:T | rs8067252 | 17 | 29263700 | 2.61E-09 | ADAP2 |
| 17:30023645-30033514 | 17:30033514:C:T | rs76954792 | 17 | 30033514 | 3.36E-09 | RP11-805L22.1 |
| 18:46306939-46346479 | 18:46343070:A:G | rs12327213 | 18 | 46343070 | 9.86E-09 | CTIF |
| 19:30277729-30328753 | 19:30314666:C:T | rs7257694 | 19 | 30314666 | 1.85E-08 | CCNE1 |
| 20:19455985-19488952 | 20:19475014:A:G | rs3827986 | 20 | 19475014 | 3.58E-08 | SLC24A3 |
| 21:35593827-35625113 | 21:35593827:A:G | rs28451064 | 21 | 35593827 | 1.00E-09 | AP000320.7:AP000318.2 |
| 22:40528220-40720963 | 22:40544337:C:T | rs5995825 | 22 | 40544337 | 1.24E-11 | TNRC6B |

TAA, thoracic aortic aneurysm; GWAS, genome-wide association study; MTAG, multi-trait analysis of GWAS; chr, chromosome; pos, position
