# Supplementary material for: Dissecting Shared Genetic Architecture of Thoracic Aortic Aneurysm and Aortic Related Traits and Identifying SplA/Ryanodine Receptor Domain and SOCS Box Containing 1 Involved in Smooth Muscle Phenotype Switching and Cell Senescence Through Alternative Splicing
Source: FASEB J. 2025 Nov 18;39(22):e71117. doi: 10.1096/fj.202502457R (PMC12637301; doi:10.1096/fj.202502457R)
Supplement: Supplementary file 7 — Table S7: fsb271117‐sup‐0007‐TableS7.docx. [file FSB2-39-e71117-s019.docx]

| **Supplemental Table S7. Information of verified genomic loci of MTAG-TAA with N-GWAMA** | | | | | | |
| --- | --- | --- | --- | --- | --- | --- |
| **Genomic locus** | **rsID** | **Chr** | **Position** | ***P* of MTAG** | ***P* of N-GWAMA** | **Nearest protein-coding gene** |
| 1:9434969-9443971 | rs2871651 | 1 | 9434969 | 3.80E-14 | 1.01E-13 | SPSB1 |
| 2:19693806-19772505 | rs824510 | 2 | 19725556 | 2.01E-36 | 1.18E-23 | OSR1 |
| 2:164752160-164930382 | rs16849225 | 2 | 164906820 | 1.88E-16 | 4.16E-12 | FIGN |
| 2:238210049-238233483 | rs12052878 | 2 | 238227594 | 5.26E-12 | 1.11E-12 | COL6A3 |
| 3:14818716-14928729 | rs73028182 | 3 | 14863636 | 5.45E-14 | 2.65E-12 | FGD5 |
| 3:41749669-42159179 | rs9847006 | 3 | 41755359 | 4.48E-20 | 1.64E-20 | ULK4 |
| 3:58049639-58199669 | rs9817209 | 3 | 58146711 | 5.38E-16 | 4.81E-16 | FLNB |
| 3:128198980-128682169 | rs62270945 | 3 | 128201889 | 3.50E-20 | 1.11E-09 | GATA2 |
| 3:186947411-187018653 | rs698083 | 3 | 186997742 | 4.69E-16 | 8.96E-24 | MASP1 |
| 4:81164723-81202048 | rs36034102 | 4 | 81202048 | 3.85E-08 | 2.18E-10 | FGF5 |
| 4:146756490-146821725 | rs1979974 | 4 | 146800815 | 2.63E-11 | 2.97E-10 | ZNF827 |
| 4:174656889-174690452 | rs67846163 | 4 | 174656889 | 1.55E-19 | 1.34E-10 | RANP6 |
| 5:81716027-81896198 | rs2897603 | 5 | 81723109 | 3.37E-12 | 7.16E-10 | ATP6AP1L |
| 5:95162219-95776105 | rs4077816 | 5 | 95582494 | 2.58E-56 | 1.20E-30 | ELL2 |
| 5:121942164-122642381 | rs17470137 | 5 | 122531347 | 2.51E-20 | 8.42E-15 | PRDM6 |
| 5:173272518-173385908 | rs6894235 | 5 | 173285545 | 3.64E-08 | 3.93E-09 | CPEB4 |
| 6:36618821-36649593 | rs3176326 | 6 | 36647289 | 3.13E-10 | 3.26E-10 | CDKN1A |
| 6:143437445-143719278 | rs1570350 | 6 | 143592386 | 2.29E-20 | 4.33E-15 | AIG1 |
| 7:73293811-73567718 | rs6974735 | 7 | 73428222 | 6.84E-80 | 1.21E-54 | ELN |
| 7:84875267-85177938 | rs1583081 | 7 | 85034227 | 3.12E-29 | 1.68E-23 | SEMA3D |
| 8:8088230-8922464 | rs1533059 | 8 | 8684953 | 1.68E-11 | 1.14E-12 | MFHAS1 |
| 8:9708433-11878338 | rs4840467 | 8 | 10079637 | 2.90E-12 | 2.57E-12 | MSRA |
| 8:75540855-75788406 | rs2570182 | 8 | 75774738 | 2.25E-15 | 1.03E-15 | PI15 |
| 8:108286782-108536949 | rs7845785 | 8 | 108294144 | 3.97E-13 | 3.33E-11 | ANGPT1 |
| 8:124541280-124615765 | rs34557926 | 8 | 124607159 | 1.74E-23 | 3.70E-15 | FBXO32 |
| 9:127766897-127988241 | rs11793512 | 9 | 127820351 | 2.57E-09 | 7.36E-09 | SCAI |
| 10:18506911-18535616 | rs2255266 | 10 | 18514999 | 6.38E-10 | 1.24E-08 | CACNB2 |
| 10:64874754-65400080 | rs7084569 | 10 | 64876554 | 4.67E-13 | 7.99E-09 | NRBF2 |
| 10:95892659-97039458 | rs71482305 | 10 | 96119130 | 3.66E-24 | 4.44E-25 | NOC3L |
| 11:17498057-17498057 | rs77889556 | 11 | 17498057 | 7.99E-12 | 1.32E-11 | ABCC8 |
| 11:69791952-70055721 | rs875107 | 11 | 70005374 | 5.15E-20 | 8.38E-14 | ANO1 |
| 11:130266117-130307440 | rs747249 | 11 | 130271647 | 5.65E-12 | 3.91E-14 | ADAMTS8 |
| 12:21859049-22047455 | rs2307024 | 12 | 22005003 | 2.22E-20 | 3.40E-13 | ABCC9 |
| 12:57502981-57533690 | rs10876963 | 12 | 57514554 | 6.08E-09 | 9.22E-12 | STAT6 |
| 12:62571546-62825851 | rs56059115 | 12 | 62763952 | 1.43E-17 | 1.43E-12 | USP15 |
| 13:22852274-22906527 | rs7994761 | 13 | 22871446 | 4.49E-49 | 8.37E-35 | MTND3P1 |
| 14:94423922-94535823 | rs4905134 | 14 | 94459845 | 1.23E-19 | 3.76E-20 | OTUB2 |
| 15:48691494-48944563 | rs1848050 | 15 | 48862043 | 1.25E-23 | 9.32E-24 | FBN1 |
| 15:71587373-71703643 | rs1441358 | 15 | 71612514 | 5.43E-18 | 3.16E-15 | THSD4 |
| 15:78942349-79099145 | rs12906653 | 15 | 79052580 | 2.53E-09 | 1.93E-12 | ADAMTS7 |
| 16:55907600-56334990 | rs959935 | 16 | 56229578 | 7.50E-11 | 2.70E-08 | GNAO1 |
| 16:66709863-72927031 | rs77870048 | 16 | 69965021 | 2.12E-42 | 9.65E-41 | WWP2 |
| 16:88966667-89032631 | rs16965180 | 16 | 88989862 | 7.82E-20 | 1.15E-11 | CBFA2T3 |
| 17:1931965-2333758 | rs1532292 | 17 | 2097483 | 4.83E-24 | 5.31E-13 | SMG6 |
| 17:12157215-12194059 | rs7215383 | 17 | 12182246 | 1.16E-25 | 2.39E-19 | ZNF18 |
| 17:30023645-30033514 | rs76954792 | 17 | 30033514 | 3.36E-09 | 3.33E-10 | COPR5 |
| 18:46306939-46346479 | rs12327213 | 18 | 46343070 | 9.86E-09 | 3.37E-11 | CTIF |
| 19:30277729-30328753 | rs7257694 | 19 | 30314666 | 1.85E-08 | 2.24E-08 | CCNE1 |
| 21:35593827-35625113 | rs28451064 | 21 | 35593827 | 1.00E-09 | 1.13E-09 | SLC5A3 |
| 22:40528220-40720963 | rs5995825 | 22 | 40544337 | 1.24E-11 | 3.73E-11 | TNRC6B |

GWAS, genome-wide association study; MTAG, multi-trait analysis of GWAS; chr, chromosome; pos, position
