# Supplementary material for: Dissecting Shared Genetic Architecture of Thoracic Aortic Aneurysm and Aortic Related Traits and Identifying SplA/Ryanodine Receptor Domain and SOCS Box Containing 1 Involved in Smooth Muscle Phenotype Switching and Cell Senescence Through Alternative Splicing
Source: FASEB J. 2025 Nov 18;39(22):e71117. doi: 10.1096/fj.202502457R (PMC12637301; doi:10.1096/fj.202502457R)
Supplement: Supplementary file 8 — Table S8: fsb271117‐sup‐0008‐TableS8.docx. [file FSB2-39-e71117-s011.docx]

**Supplemental Table S8. Information of the results of multi-trait colocalization analysis**

| **Genomic Locus** | **Candidate SNP** | **Traits** | ***P*_A_** | ***P*_R_** | ***P*_A_×*P*_R_** | **Nearest protein-coding gene** |
| --- | --- | --- | --- | --- | --- | --- |
| 2:238210049-238233483 | rs12052878 | AAdiameter, DAdiameter | 0.9863 | 1 | 0.9863 | COL6A3 |
| 4:81164723-81202048 | rs12509595 | AAdiameter, DAdiameter | 0.9835 | 0.9969 | 0.98045115 | FGF5 |
| 3:186947411-187018653 | rs698083 | AAdiameter, DAdiameter | 0.9672 | 1 | 0.9672 | MASP1 |
| 1:9434969-9443971 | rs9662255 | AAdiameter, DAdiameter | 0.9683 | 0.996 | 0.9644268 | SPSB1 |
| 3:58049639-58199669 | rs56004178 | AAdiameter, DAdiameter | 0.9558 | 1 | 0.9558 | FLNB |
| 8:75540855-75788406 | rs2570182 | AAdiameter, DAdiameter | 0.8026 | 0.9984 | 0.80131584 | PI15 |
| 8:8088230-8922464 | rs11784052 | AAdiameter, DAdiameter | 0.7964 | 0.9692 | 0.77187088 | MFHAS1 |
| 3:41749669-42159179 | rs9847006 | AAdiameter, DAdiameter | 0.7466 | 0.9987 | 0.74562942 | ULK4 |
| 5:95162219-95776105 | rs4077816 | TAA, AAdiameter | 0.7107 | 0.985 | 0.7000395 | ELL2 |
| 7:84875267-85177938 | rs1583081 | AAdiameter, DAdiameter | 0.8213 | 0.8428 | 0.69219164 | SEMA3D |
| 8:9708433-11878338 | rs4394398 | AAdiameter, DAdiameter | 0.771 | 0.893 | 0.688503 | MSRA |
| 3:14818716-14928729 | rs11712199 | AAdiameter, DAdiameter | 0.6827 | 0.6874 | 0.46928798 | FGD5 |
| 2:164752160-164930382 | rs16849225 | AAdiameter, DAdiameter | 0.6564 | 0.6914 | 0.45383496 | FIGN |
| 10:95892659-97039458 | rs10882399 | DAdiameter, DAmax, DAmin | 0.5883 | 0.6593 | 0.38786619 | NOC3L |
| 7:73293811-73567718 | rs6974735 | TAA, AAdiameter, DAdiameter | 0.4374 | 0.5007 | 0.21900618 | ELN |

TAA, thoracic aortic aneurysm; AAdiameter, ascending aortic diameter; DAdiameter, descending aortic diameter; AAmax, ascending aortic max area; DAmax, descending aortic max area; AAmin, ascending aortic minimum area; DAmin, descending aortic minimum area; AAdis, ascending aortic distensibility; DAdis, descending aortic diameter; AAstrain, ascending aortic strain; DAstrain, descending aortic strain; *P*_A_, posterior probability; *P*_R_, regional probability.
