# Supplementary material for: Dissecting Shared Genetic Architecture of Thoracic Aortic Aneurysm and Aortic Related Traits and Identifying SplA/Ryanodine Receptor Domain and SOCS Box Containing 1 Involved in Smooth Muscle Phenotype Switching and Cell Senescence Through Alternative Splicing
Source: FASEB J. 2025 Nov 18;39(22):e71117. doi: 10.1096/fj.202502457R (PMC12637301; doi:10.1096/fj.202502457R)
Supplement: Supplementary file 9 — Table S9: fsb271117‐sup‐0009‐TableS9.docx. [file FSB2-39-e71117-s016.docx]

**Supplemental Table S9. Information of MR-JTI results**

| **Gene** | **Symbol** | **β** | **95% lower CI** | **95% upper CI** | ***P*** |
| --- | --- | --- | --- | --- | --- |
| ENSG00000168038 | ULK4 | 0.722494739 | 0.349847759 | 0.798378444 | 5.23E-07 |
| ENSG00000061455 | PRDM6 | 0.733567207 | 0.472093051 | 1.089930086 | 7.22E-07 |
| ENSG00000135655 | USP15 | 0.682696491 | 0.306367984 | 0.954388682 | 1.37E-04 |
| ENSG00000253893 | FAM85B | -0.768602194 | -1.098952467 | -0.323707067 | 3.22E-04 |
| ENSG00000121989 | ACVR2A | 0.599789827 | 0.23126611 | 0.971116503 | 1.45E-03 |
| ENSG00000169554 | ZEB2 | 0.616803768 | 0.200598704 | 0.97578324 | 2.94E-03 |
| ENSG00000010017 | RANBP9 | 0.471230454 | 0.134247076 | 0.69731214 | 3.80E-03 |
| ENSG00000123384 | LRP1 | 0.554852576 | 0.16755431 | 0.885301257 | 4.04E-03 |
| ENSG00000164532 | TBX20 | 0.360266967 | 0.094517808 | 0.58906527 | 6.74E-03 |
| ENSG00000188994 | ZNF292 | 0.51911843 | 0.122154975 | 0.835661139 | 8.51E-03 |
| ENSG00000134917 | ADAMTS8 | 0.41536514 | 0.094520796 | 0.688693187 | 9.78E-03 |
| ENSG00000187720 | THSD4 | -0.403693914 | -0.830070591 | -0.110969022 | 1.03E-02 |
| ENSG00000066382 | MPPED2 | 0.52006023 | 0.100968351 | 1.020414654 | 1.68E-02 |
| ENSG00000225921 | NOL7 | 0.481501633 | 0.073863756 | 0.751793276 | 1.70E-02 |
| ENSG00000251409 | AC008592.4 | 0.117048382 | 0.028364385 | 0.29306588 | 1.73E-02 |
| ENSG00000173852 | DPY19L1 | 0.361898019 | 0.026990524 | 0.60869826 | 3.22E-02 |
| ENSG00000093167 | LRRFIP2 | 0.363294529 | 0.020514326 | 0.557183635 | 3.49E-02 |
| ENSG00000197321 | SVIL | 0.270587681 | 0.01993182 | 0.54986586 | 3.51E-02 |
| ENSG00000127129 | EDN2 | -0.404486326 | -0.817136974 | -0.023763303 | 3.78E-02 |
| ENSG00000198743 | SLC5A3 | 0.179202676 | 0.008086847 | 0.365509165 | 4.05E-02 |
| ENSG00000243927 | MRPS6 | 0.181966514 | 0.006152816 | 0.368093671 | 4.27E-02 |
| ENSG00000116194 | ANGPTL1 | 0.324048462 | 0.011262024 | 0.787640964 | 4.37E-02 |
| ENSG00000136044 | APPL2 | -0.169681371 | -0.332985621 | -0.004350448 | 4.42E-02 |

β, expression beta of corresponding gene
