# Supplementary material for: Dissecting Shared Genetic Architecture of Thoracic Aortic Aneurysm and Aortic Related Traits and Identifying SplA/Ryanodine Receptor Domain and SOCS Box Containing 1 Involved in Smooth Muscle Phenotype Switching and Cell Senescence Through Alternative Splicing
Source: FASEB J. 2025 Nov 18;39(22):e71117. doi: 10.1096/fj.202502457R (PMC12637301; doi:10.1096/fj.202502457R)
Supplement: Supplementary file 10 — Table S10: fsb271117‐sup‐0010‐TableS10.docx. [file FSB2-39-e71117-s017.docx]

**Supplemental Table S10. Information of the candidate genes' expression levels in gene expression array of TAA human and RNA-seq of BAPN-induced TAA mice**

| **Gene** | **Control mean value** | **TAA mean value** | **Adjusted *P* value** |
| --- | --- | --- | --- |
| **Human** |  |  |  |
| ACVR2A | 6.81 | 6.73 | 4.01E-01 |
| ADAMTS8 | 7.43 | 7.37 | 7.07E-01 |
| ANGPTL1 | 5.97 | 5.43 | 2.38E-02 |
| APPL2 | 7.71 | 7.89 | 4.02E-02 |
| COL6A3 | 8.29 | 8.99 | 1.89E-06 |
| EDN2 | 6.44 | 6.29 | 3.57E-02 |
| ELL2 | 8.93 | 8.52 | 2.91E-03 |
| ELN | 11.06 | 11.22 | 4.28E-01 |
| FGD5 | 5.72 | 6.18 | 2.24E-05 |
| FGF5 | 3.37 | 3.35 | 9.03E-01 |
| FIGN | 6.78 | 6.77 | 9.46E-01 |
| FLNB | 8.00 | 7.76 | 4.28E-02 |
| LRP1 | 10.14 | 10.49 | 3.05E-04 |
| LRRFIP2 | 7.62 | 7.35 | 3.87E-02 |
| MASP1 | 6.45 | 6.87 | 2.01E-02 |
| MFHAS1 | 7.40 | 7.60 | 1.15E-02 |
| MPPED2 | 8.42 | 7.90 | 1.38E-02 |
| MRPS6 | 9.09 | 9.07 | 8.06E-01 |
| MSRA | 6.77 | 6.60 | 6.12E-02 |
| NOC3L | 4.88 | 5.06 | 1.10E-01 |
| NOL7 | 7.61 | 7.49 | 2.05E-01 |
| PI15 | 3.98 | 3.75 | 9.28E-02 |
| RANBP9 | 8.96 | 8.60 | 2.00E-02 |
| SEMA3D | 7.66 | 5.77 | 4.90E-06 |
| SLC5A3 | 10.78 | 10.99 | 2.42E-01 |
| SPSB1 | 8.07 | 8.63 | 7.29E-05 |
| SVIL | 10.44 | 10.27 | 1.98E-01 |
| TBX20 | 5.65 | 6.13 | 1.58E-02 |
| THSD4 | 7.72 | 7.09 | 1.16E-03 |
| ULK4 | 5.57 | 5.63 | 5.73E-01 |
| USP15 | 7.27 | 7.11 | 1.34E-01 |
| **Mouse** |  |  |  |
| ZEB2 | 8.67 | 8.90 | 6.78E-02 |
| ACVR2A | 7.57344 | 7.2189425 | 1.14E-02 |
| ADAMTS8 | 5.97585 | 7.2940575 | 4.40E-07 |
| ANGPTL1 | 6.0417375 | 6.86766 | 1.77E-03 |
| APPL2 | 7.6853825 | 7.438455 | 1.85E-01 |
| COL6A3 | 10.34875 | 10.2772 | 3.54E-01 |
| DPY19L1 | 8.1674675 | 8.2922675 | 1.76E-01 |
| EDN2 | 4.9901825 | 5.113835 | 4.72E-01 |
| ELL2 | 8.200615 | 8.182435 | 8.82E-01 |
| ELN | 12.155925 | 12.321575 | 4.84E-02 |
| FGD5 | 6.6537825 | 6.38165 | 1.04E-01 |
| FGF5 | 4.60455 | 4.8135675 | 2.47E-01 |
| FIGN | 5.29045 | 5.25E+00 | 8.39E-01 |
| FLNB | 8.8006825 | 9.169545 | 3.84E-03 |
| LRP1 | 9.391125 | 9.5017325 | 2.03E-01 |
| LRRFIP2 | 7.5618775 | 7.280885 | 2.29E-02 |
| MASP1 | 4.7303525 | 4.6543 | 6.77E-01 |
| MFHAS1 | 6.6883025 | 6.6098125 | 4.72E-01 |
| MPPED2 | 6.7224675 | 7.02892 | 8.96E-03 |
| MRPS6 | 8.1326275 | 7.898015 | 3.48E-01 |
| MSRA | 6.370855 | 6.3793325 | 8.89E-01 |
| NOC3L | 6.656155 | 6.9220875 | 3.29E-03 |
| NOL7 | 7.8403825 | 7.8014375 | 7.18E-01 |
| PI15 | 10.31155 | 9.48E+00 | 2.39E-03 |
| PRDM6 | 8.9925525 | 8.92E+00 | 5.66E-01 |
| RANBP9 | 9.60805 | 9.28E+00 | 4.33E-02 |
| SEMA3D | 7.38141 | 7.66E+00 | 2.62E-01 |
| SPSB1 | 6.7312075 | 7.0311725 | 6.69E-03 |
| SVIL | 8.3859175 | 8.36E+00 | 8.23E-01 |
| TBX20 | 7.7669475 | 7.58E+00 | 6.17E-01 |
| THSD4 | 8.0504825 | 7.73E+00 | 6.35E-02 |
| ULK4 | 5.62751 | 5.5121125 | 4.09E-01 |
| USP15 | 8.1717025 | 8.30E+00 | 3.53E-02 |
| ZEB2 | 7.7986975 | 7.75E+00 | 6.40E-01 |

31 control and 22 TAA patients were used in the human dataset, 3 control and 3 BAPN-induced mice were used in the mouse dataset. The *P* values were adjusted with Bonferroni method.
