# Supplementary material for: Dissecting Shared Genetic Architecture of Thoracic Aortic Aneurysm and Aortic Related Traits and Identifying SplA/Ryanodine Receptor Domain and SOCS Box Containing 1 Involved in Smooth Muscle Phenotype Switching and Cell Senescence Through Alternative Splicing
Source: FASEB J. 2025 Nov 18;39(22):e71117. doi: 10.1096/fj.202502457R (PMC12637301; doi:10.1096/fj.202502457R)
Supplement: Supplementary file 11 — Table S11: fsb271117‐sup‐0011‐TableS11.docx. [file FSB2-39-e71117-s009.docx]

**Supplemental Table S11. Gene Ontology analysis results for the co-expression analysis of SPSB1.**

| **Ontology** | **ID** | **Description** | **Gene ratio** | **Adjusted *p* value** | **Q value** |
| --- | --- | --- | --- | --- | --- |
| BP | GO:0002181 | cytoplasmic translation | 30/961 | 1.33E-06 | 1.17E-06 |
| BP | GO:0043161 | proteasome-mediated ubiquitin-dependent protein catabolic process | 56/961 | 5.64E-06 | 2.47E-06 |
| BP | GO:0008380 | RNA splicing | 56/961 | 2.70E-05 | 7.89E-06 |
| BP | GO:0043484 | regulation of RNA splicing | 29/961 | 2.00E-04 | 4.39E-05 |
| BP | GO:0007015 | actin filament organization | 51/961 | 1.01E-03 | 1.78E-04 |
| BP | GO:0032386 | regulation of intracellular transport | 40/961 | 2.78E-03 | 3.93E-04 |
| BP | GO:0031647 | regulation of protein stability | 39/961 | 3.14E-03 | 3.93E-04 |
| BP | GO:0016032 | viral process | 47/961 | 4.09E-03 | 4.49E-04 |
| BP | GO:0043244 | regulation of protein-containing complex disassembly | 21/961 | 5.08E-03 | 4.95E-04 |
| BP | GO:1901879 | regulation of protein depolymerization | 17/961 | 6.67E-03 | 5.85E-04 |
| CC | GO:0005925 | focal adhesion | 80/1004 | 1.89E-22 | 7.38E-23 |
| CC | GO:0030055 | cell-substrate junction | 81/1004 | 2.01E-22 | 7.38E-23 |
| CC | GO:0022626 | cytosolic ribosome | 27/1004 | 1.64E-08 | 4.03E-09 |
| CC | GO:0043292 | contractile fiber | 39/1004 | 1.09E-07 | 1.81E-08 |
| CC | GO:0005840 | ribosome | 39/1004 | 1.23E-07 | 1.81E-08 |
| CC | GO:0032432 | actin filament bundle | 20/1004 | 9.22E-07 | 1.13E-07 |
| CC | GO:0030016 | myofibril | 36/1004 | 1.79E-06 | 1.88E-07 |
| CC | GO:0044391 | ribosomal subunit | 31/1004 | 3.39E-06 | 3.11E-07 |
| CC | GO:0001725 | stress fiber | 18/1004 | 4.64E-06 | 3.41E-07 |
| CC | GO:0097517 | contractile actin filament bundle | 18/1004 | 4.64E-06 | 3.41E-07 |
| MF | GO:0045296 | cadherin binding | 53/987 | 7.77E-10 | 6.96E-10 |
| MF | GO:0003779 | actin binding | 56/987 | 9.99E-07 | 4.48E-07 |
| MF | GO:0003735 | structural constituent of ribosome | 28/987 | 1.80E-04 | 5.38E-05 |
| MF | GO:0048306 | calcium-dependent protein binding | 15/987 | 1.40E-02 | 3.14E-03 |
| MF | GO:0008135 | translation factor activity, RNA binding | 15/987 | 3.46E-02 | 6.21E-03 |
| MF | GO:0016859 | cis-trans isomerase activity | 10/987 | 4.88E-02 | 7.29E-03 |
| MF | GO:0003714 | transcription corepressor activity | 24/987 | 9.62E-02 | 1.23E-02 |
| MF | GO:0051015 | actin filament binding | 25/987 | 1.11E-01 | 1.25E-02 |
| MF | GO:0003755 | peptidyl-prolyl cis-trans isomerase activity | 9/987 | 1.49E-01 | 1.48E-02 |
| MF | GO:0005178 | integrin binding | 20/987 | 1.82E-01 | 1.54E-02 |
