# Supplementary material for: Dissecting Shared Genetic Architecture of Thoracic Aortic Aneurysm and Aortic Related Traits and Identifying SplA/Ryanodine Receptor Domain and SOCS Box Containing 1 Involved in Smooth Muscle Phenotype Switching and Cell Senescence Through Alternative Splicing
Source: FASEB J. 2025 Nov 18;39(22):e71117. doi: 10.1096/fj.202502457R (PMC12637301; doi:10.1096/fj.202502457R)
Supplement: Supplementary file 12 — Table S12: fsb271117‐sup‐0012‐TableS12.docx. [file FSB2-39-e71117-s001.docx]

**Supplemental Table S12. Enrichment analysis results for the data-independent acquisition (DIA)-based quantitative proteomic analysis of SPSB1-knockdown and control aortic SMC**

| **ID** | **Term** | **Category** | **Protein ratio** | **Adjusted *p* value** | **Q value** |
| --- | --- | --- | --- | --- | --- |
| **GO analysis** |  |  |  |  |  |
| GO:0019899 | enzyme binding | MF | 30/404 | 2.68E-25 | 5.47E-23 |
| GO:0042803 | protein homodimerization activity | MF | 42/824 | 2.50E-28 | 6.02E-26 |
| GO:0045296 | cadherin binding | MF | 34/368 | 1.15E-31 | 3.81E-29 |
| GO:0003723 | RNA binding | MF | 119/3562 | 3.46E-59 | 2.62E-56 |
| GO:0042802 | identical protein binding | MF | 118/1981 | 4.81E-86 | 5.10E-83 |
| GO:0005634 | nucleus | CC | 267/14933 | 2.12E-77 | 1.88E-74 |
| GO:0005654 | nucleoplasm | CC | 233/8518 | 3.67E-102 | 4.86E-99 |
| GO:0005737 | cytoplasm | CC | 307/12155 | 4.11E-130 | 7.26E-127 |
| GO:0070062 | extracellular exosome | CC | 211/2117 | 3.93E-204 | 1.04E-200 |
| GO:0005829 | cytosol | CC | 368/9748 | 2.67E-220 | 1.42E-216 |
| GO:0045071 | negative regulation of viral genome replication | BP | 14/44 | 6.56E-22 | 1.05E-19 |
| GO:0045944 | positive regulation of transcription by RNA polymerase II | BP | 48/1330 | 1.22E-25 | 2.59E-23 |
| GO:0016477 | cell migration | BP | 28/267 | 7.25E-28 | 1.67E-25 |
| GO:0045087 | innate immune response | BP | 46/933 | 2.74E-30 | 7.64E-28 |
| GO:0051607 | defense response to virus | BP | 35/407 | 1.79E-31 | 5.57E-29 |
| **KEGG analysis** | |  |  |  |  |
| hsa04142 | Cellular Processes | Lysosome | 24/175 | 2.73E-07 | 8.78E-05 |
| hsa05165 | Human Diseases | Human papillomavirus infection | 44/478 | 6.53E-07 | 1.05E-04 |
| hsa04820 | Cellular Processes | Cytoskeleton in muscle cells | 33/320 | 1.51E-06 | 1.62E-04 |
| hsa04512 | Environmental Information Processing | ECM-receptor interaction | 18/128 | 5.99E-06 | 4.80E-04 |
| hsa00230 | Metabolism | Purine metabolism | 20/176 | 4.63E-05 | 2.97E-03 |
| hsa01232 | Metabolism | Nucleotide metabolism | 15/119 | 1.28E-04 | 6.85E-03 |
| hsa04510 | Cellular Processes | Focal adhesion | 27/308 | 2.28E-04 | 1.04E-02 |
| hsa00280 | Metabolism | Valine, leucine and isoleucine degradation | 10/73 | 8.74E-04 | 3.51E-02 |
| hsa04148 | Cellular Processes | Efferocytosis | 20/229 | 1.51E-03 | 5.39E-02 |
| hsa00511 | Metabolism | Other glycan degradation | 5/24 | 2.77E-03 | 8.88E-02 |
