# Supplementary material for: Dissecting Shared Genetic Architecture of Thoracic Aortic Aneurysm and Aortic Related Traits and Identifying SplA/Ryanodine Receptor Domain and SOCS Box Containing 1 Involved in Smooth Muscle Phenotype Switching and Cell Senescence Through Alternative Splicing
Source: FASEB J. 2025 Nov 18;39(22):e71117. doi: 10.1096/fj.202502457R (PMC12637301; doi:10.1096/fj.202502457R)
Supplement: Supplementary file 13 — Table S13: fsb271117‐sup‐0013‐TableS13.docx. [file FSB2-39-e71117-s005.docx]

**Supplemental Table S13.** **Enrichment analysis results for the RNA-seq of** **SPSB1-knockdown and control aortic SMC**

| **ID** | **Term** | **Category** | **Gene ratio** | **Adjusted *P* value** | **Q value** |
| --- | --- | --- | --- | --- | --- |
| **GO enrichment** | |  |  |  |  |
| GO:0006355 | regulation of DNA-templated transcription | BP | 119/1605 | 1.44E-05 | 2.05E-03 |
| GO:0006357 | regulation of transcription by RNA polymerase II | BP | 111/1637 | 8.65E-04 | 4.21E-02 |
| GO:0045944 | positive regulation of transcription by RNA polymerase II | BP | 96/1280 | 6.48E-05 | 6.41E-03 |
| GO:0007165 | signal transduction | BP | 95/2488 | 9.99E-01 | 1.00E+00 |
| GO:0000122 | negative regulation of transcription by RNA polymerase II | BP | 85/1048 | 1.09E-05 | 1.74E-03 |
| GO:0006974 | DNA damage response | BP | 75/613 | 1.21E-12 | 7.53E-10 |
| GO:0016310 | phosphorylation | BP | 69/720 | 2.59E-07 | 7.68E-05 |
| GO:0015031 | protein transport | BP | 68/718 | 4.86E-07 | 1.26E-04 |
| GO:0006281 | DNA repair | BP | 67/461 | 5.77E-15 | 5.14E-12 |
| GO:0030154 | cell differentiation | BP | 67/1061 | 3.44E-02 | 2.49E-01 |
| GO:0007049 | cell cycle | BP | 66/697 | 7.19E-07 | 1.60E-04 |
| GO:0006915 | apoptotic process | BP | 59/809 | 2.99E-03 | 8.37E-02 |
| GO:0006468 | protein phosphorylation | BP | 55/596 | 1.26E-05 | 1.91E-03 |
| GO:0045893 | positive regulation of DNA-templated transcription | BP | 55/865 | 4.63E-02 | 2.49E-01 |
| GO:0045892 | negative regulation of DNA-templated transcription | BP | 52/684 | 2.22E-03 | 7.87E-02 |
| GO:0005737 | cytoplasm | CC | 617/7797 | 2.68E-40 | 5.57E-37 |
| GO:0005634 | nucleus | CC | 606/7325 | 8.13E-46 | 2.54E-42 |
| GO:0016020 | membrane | CC | 538/10335 | 1.72E-01 | 3.86E-01 |
| GO:0005829 | cytosol | CC | 507/5921 | 4.09E-40 | 6.37E-37 |
| GO:0005654 | nucleoplasm | CC | 379/4320 | 3.53E-30 | 4.41E-27 |
| GO:0005886 | plasma membrane | CC | 308/6493 | 9.03E-01 | 9.25E-01 |
| GO:0005856 | cytoskeleton | CC | 152/1603 | 2.37E-14 | 1.85E-11 |
| GO:0070062 | extracellular exosome | CC | 148/2362 | 3.22E-03 | 8.61E-02 |
| GO:0005739 | mitochondrion | CC | 142/1788 | 3.90E-08 | 1.35E-05 |
| GO:0005783 | endoplasmic reticulum | CC | 118/1851 | 4.86E-03 | 1.15E-01 |
| GO:0005794 | Golgi apparatus | CC | 116/1577 | 2.62E-05 | 3.41E-03 |
| GO:0005730 | nucleolus | CC | 108/2069 | 3.65E-01 | 5.29E-01 |
| GO:0042995 | cell projection | CC | 103/1371 | 3.28E-05 | 4.01E-03 |
| GO:0005576 | extracellular region | CC | 93/2964 | 1.00E+00 | 1.00E+00 |
| GO:0043231 | intracellular membrane-bounded organelle | CC | 87/1121 | 4.36E-05 | 4.77E-03 |
| GO:0005515 | protein binding | MF | 1070/15345 | 3.89E-69 | 2.42E-65 |
| GO:0046872 | metal ion binding | MF | 329/4309 | 5.25E-16 | 5.46E-13 |
| GO:0003677 | DNA binding | MF | 188/2466 | 4.40E-09 | 1.83E-06 |
| GO:0000166 | nucleotide binding | MF | 173/2041 | 6.11E-12 | 3.47E-09 |
| GO:0003723 | RNA binding | MF | 159/1730 | 7.88E-14 | 5.46E-11 |
| GO:0016787 | hydrolase activity | MF | 156/1887 | 4.76E-10 | 2.12E-07 |
| GO:0016740 | transferase activity | MF | 153/2058 | 6.69E-07 | 1.54E-04 |
| GO:0005524 | ATP binding | MF | 147/1666 | 1.65E-11 | 7.92E-09 |
| GO:0042802 | identical protein binding | MF | 137/1914 | 1.83E-05 | 2.43E-03 |
| GO:0000978 | RNA polymerase II cis-regulatory region sequence-specific DNA binding | MF | 90/1220 | 1.92E-04 | 1.50E-02 |
| GO:0008270 | zinc ion binding | MF | 72/977 | 8.42E-04 | 4.20E-02 |
| GO:0000981 | DNA-binding transcription factor activity, RNA polymerase II-specific | MF | 72/1232 | 1.07E-01 | 3.24E-01 |
| GO:0016301 | kinase activity | MF | 69/722 | 2.87E-07 | 8.13E-05 |
| GO:0042803 | protein homodimerization activity | MF | 59/774 | 1.10E-03 | 4.85E-02 |
| GO:0003676 | nucleic acid binding | MF | 58/721 | 3.27E-04 | 2.24E-02 |
| **KEGG enrichment** | |  |  |  |  |
| hsa00230 | Metabolism | Purine metabolism | 19/135 | 2.53E-04 | 8.02E-02 |
| hsa03410 | Genetic Information Processing | Base excision repair | 9/47 | 1.24E-03 | 1.42E-01 |
| hsa03266 | Genetic Information Processing | Virion - Herpesvirus | 4/10 | 1.72E-03 | 1.42E-01 |
| hsa00240 | Metabolism | Pyrimidine metabolism | 10/59 | 1.79E-03 | 1.42E-01 |
| hsa05168 | Human Diseases | Herpes simplex virus 1 infection | 58/709 | 3.79E-03 | 2.40E-01 |
| hsa04520 | Cellular Processes | Adherens junction | 13/104 | 6.52E-03 | 3.44E-01 |
| hsa05161 | Human Diseases | Hepatitis B | 19/180 | 7.59E-03 | 3.44E-01 |
| hsa00515 | Metabolism | Mannose type O-glycan biosynthesis | 5/24 | 1.06E-02 | 3.77E-01 |
| hsa05162 | Human Diseases | Measles | 17/161 | 1.12E-02 | 3.77E-01 |
| hsa04064 | Environmental Information Processing | NF-kappa B signaling pathway | 15/137 | 1.21E-02 | 3.77E-01 |
| hsa05165 | Human Diseases | Human papillomavirus infection | 35/412 | 1.31E-02 | 3.77E-01 |
| hsa04144 | Cellular Processes | Endocytosis | 28/316 | 1.50E-02 | 3.82E-01 |
| hsa04620 | Organismal Systems | Toll-like receptor signaling pathway | 14/129 | 1.62E-02 | 3.82E-01 |
| hsa00310 | Metabolism | Lysine degradation | 9/70 | 1.83E-02 | 3.82E-01 |
| hsa04340 | Environmental Information Processing | Hedgehog signaling pathway | 8/59 | 1.90E-02 | 3.82E-01 |
| hsa04010 | Environmental Information Processing | MAPK signaling pathway | 29/337 | 1.93E-02 | 3.82E-01 |
| hsa05131 | Human Diseases | Shigellosis | 24/272 | 2.42E-02 | 4.46E-01 |
| hsa04110 | Cellular Processes | Cell cycle | 16/163 | 2.53E-02 | 4.46E-01 |
| hsa03083 | Genetic Information Processing | Polycomb repressive complex | 11/100 | 2.83E-02 | 4.59E-01 |
| hsa04140 | Cellular Processes | Autophagy - animal | 17/180 | 3.01E-02 | 4.59E-01 |
