# Supplementary figures and images for: Dissecting Shared Genetic Architecture of Thoracic Aortic Aneurysm and Aortic Related Traits and Identifying SplA/Ryanodine Receptor Domain and SOCS Box Containing 1 Involved in Smooth Muscle Phenotype Switching and Cell Senescence Through Alternative Splicing
Source: FASEB J. 2025 Nov 18;39(22):e71117. doi: 10.1096/fj.202502457R (PMC12637301; doi:10.1096/fj.202502457R)

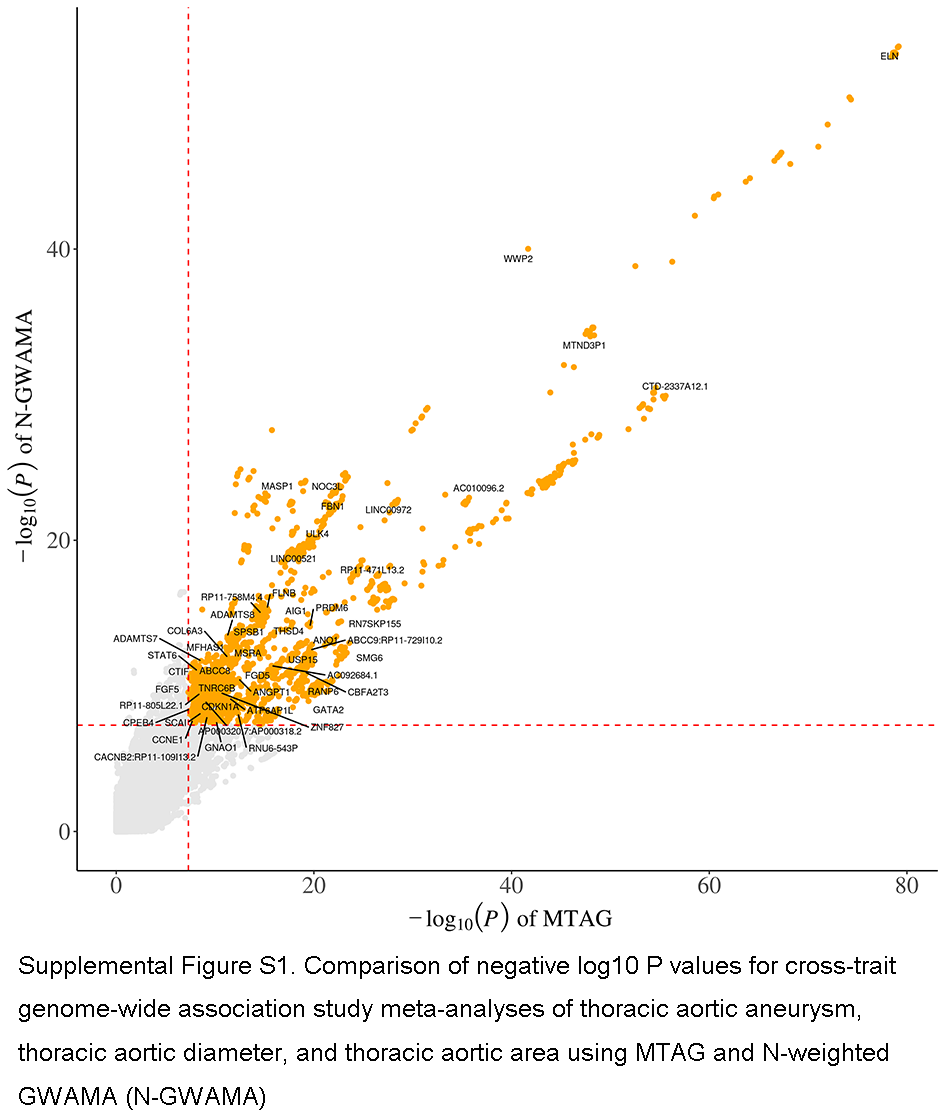

Supplement: Supplementary file 14 — Figure S1: fsb271117‐sup‐0014‐FigureS1.tif. [file FSB2-39-e71117-s002.tif]

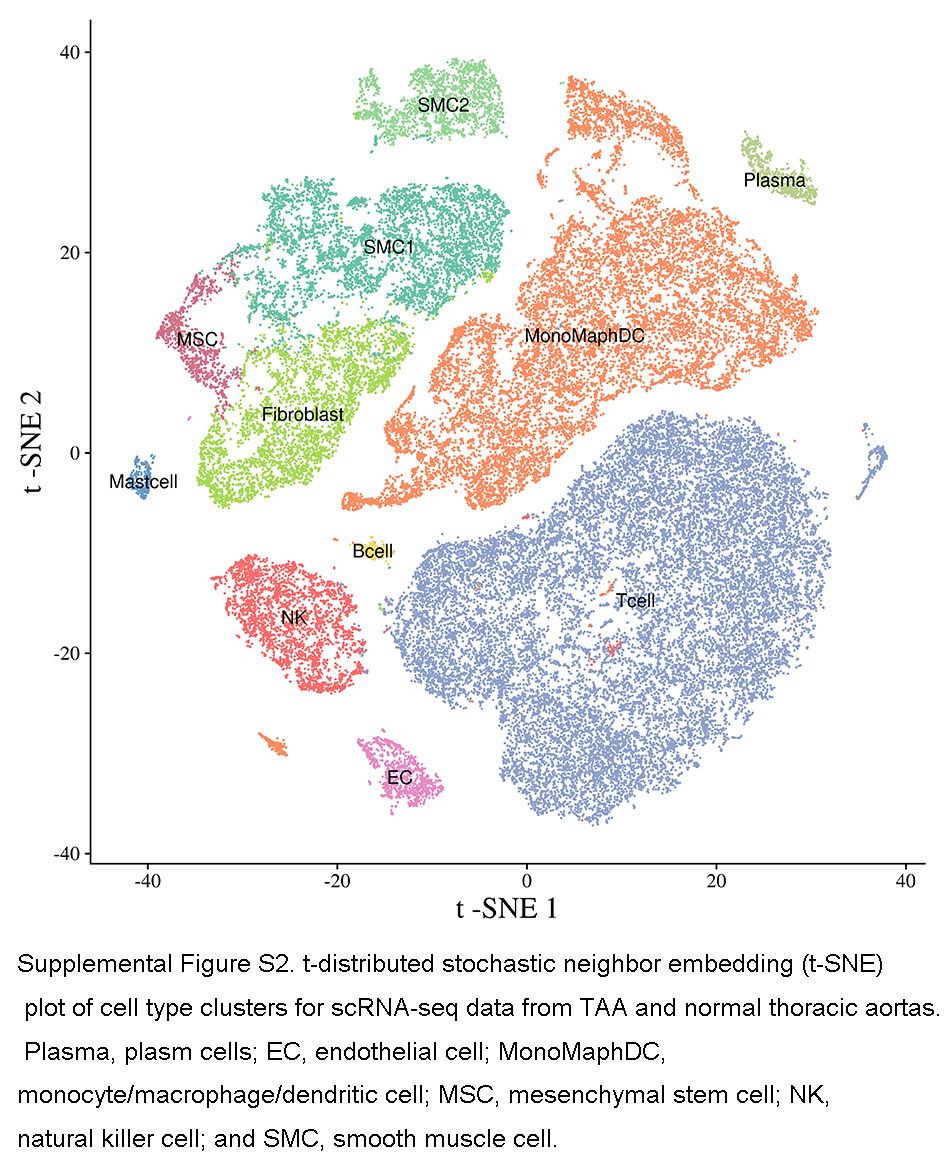

Supplement: Supplementary file 15 — Figure S2: fsb271117‐sup‐0015‐FigureS2.tif. [file FSB2-39-e71117-s006.tif]

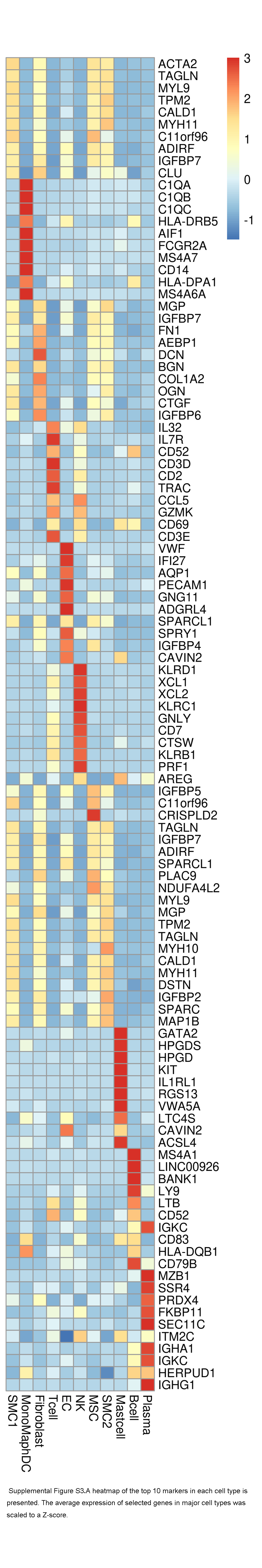

Supplement: Supplementary file 16 — Figure S3: fsb271117‐sup‐0016‐FigureS3.tif. [file FSB2-39-e71117-s014.tif]

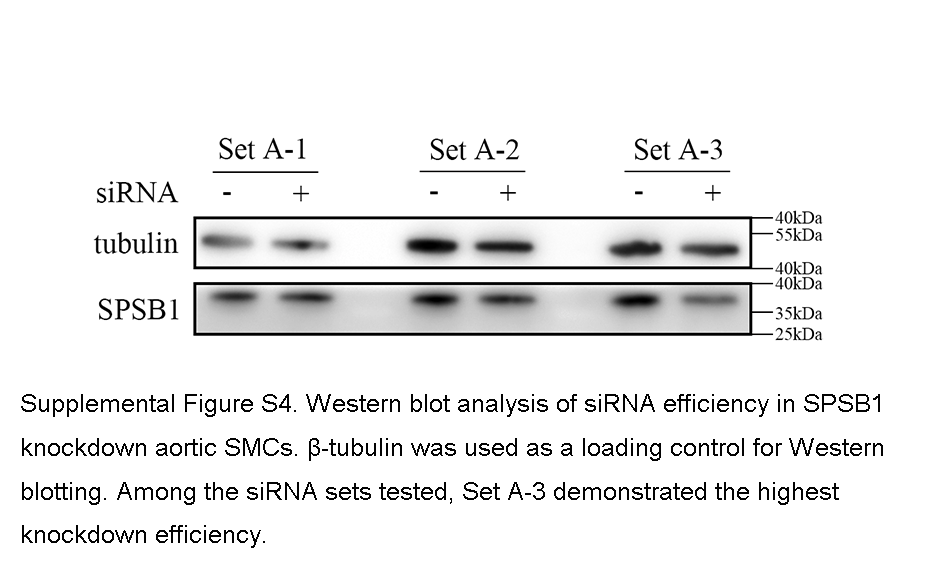

Supplement: Supplementary file 17 — Figure S4: fsb271117‐sup‐0017‐FigureS4.tif. [file FSB2-39-e71117-s018.tif]

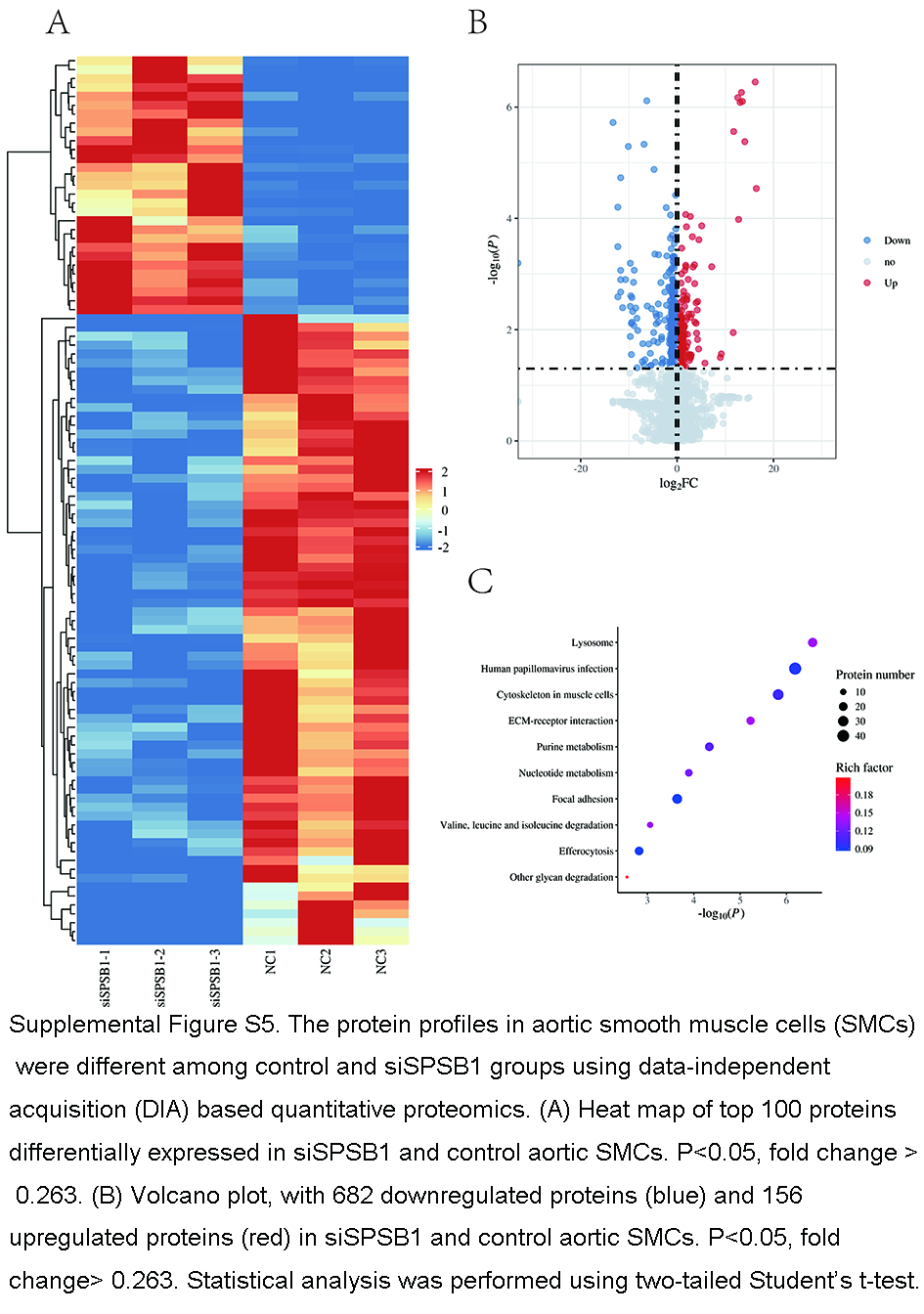

Supplement: Supplementary file 18 — Figure S5: fsb271117‐sup‐0018‐FigureS5.tif. [file FSB2-39-e71117-s007.tif]

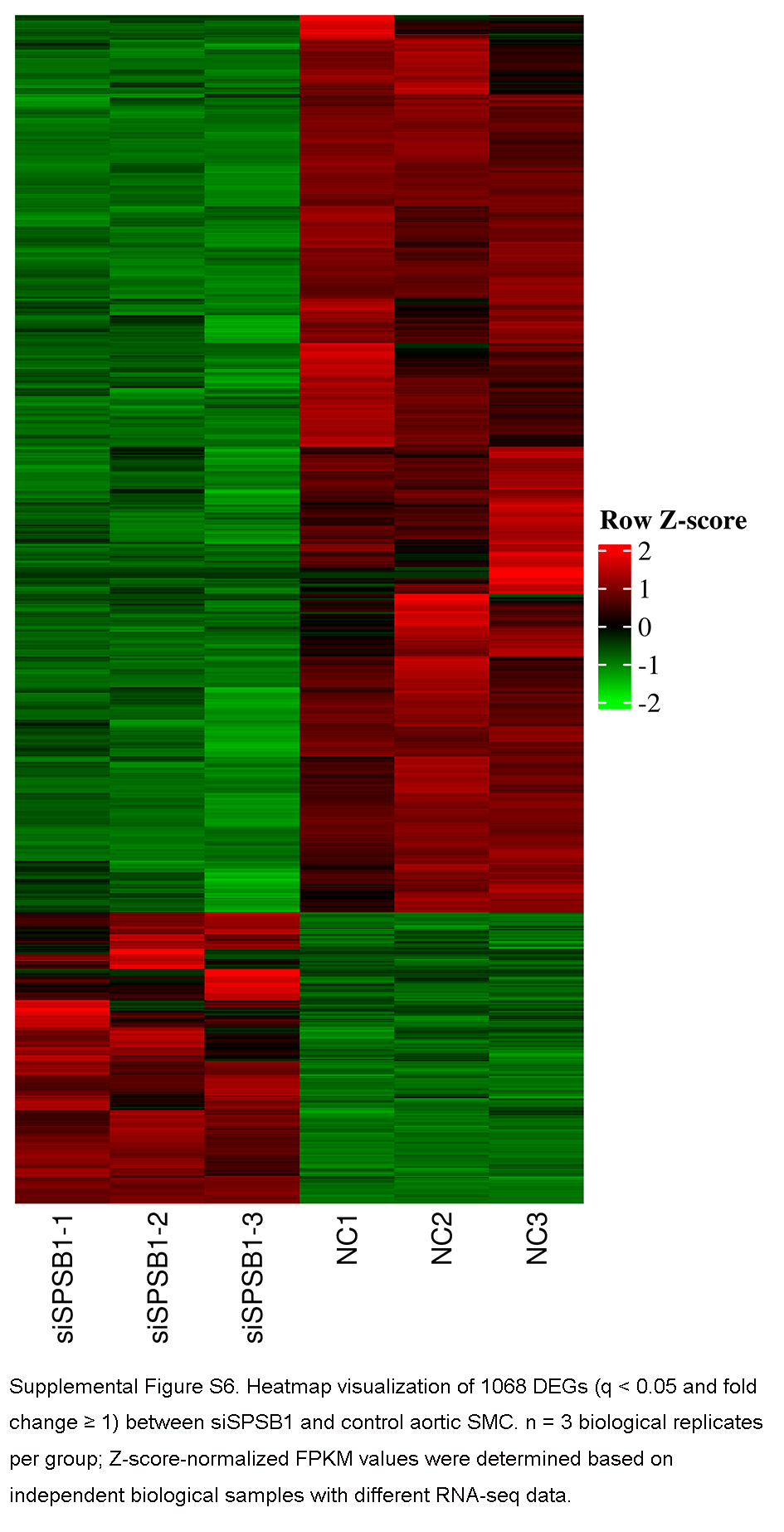

Supplement: Supplementary file 19 — Figure S6: fsb271117‐sup‐0019‐FigureS6.tif. [file FSB2-39-e71117-s003.tif]

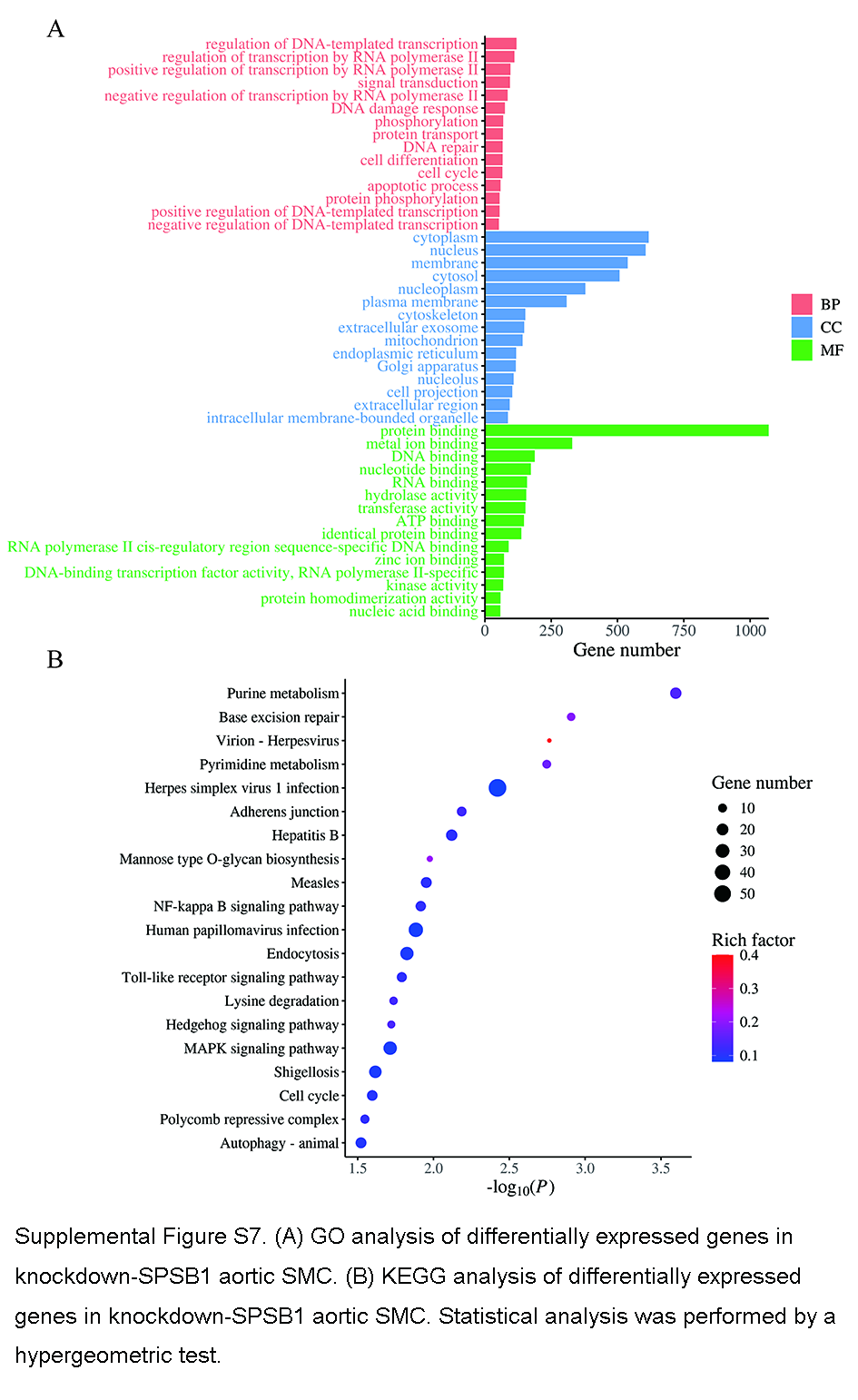

Supplement: Supplementary file 20 — Figure S7: fsb271117‐sup‐0020‐FigureS7.tif. [file FSB2-39-e71117-s013.tif]
